# *Mycobacterium bovis*: From Genotyping to Genome Sequencing

**DOI:** 10.3390/microorganisms8050667

**Published:** 2020-05-03

**Authors:** Ana M. S. Guimaraes, Cristina K. Zimpel

**Affiliations:** 1 Laboratory of Applied Research in Mycobacteria, Department of Microbiology, University of São Paulo, São Paulo 01246-904, Brazil; ckzimpel@gmail.com; 2 Department of Preventive Veterinary Medicine and Animal Health, University of São Paulo, São Paulo 01246-904, Brazil

**Keywords:** bovine tuberculosis, *Mycobacterium bovis*, genomics, WGS, genotyping

## Abstract

*Mycobacterium bovis* is the main pathogen of bovine, zoonotic, and wildlife tuberculosis. Despite the existence of programs for bovine tuberculosis (bTB) control in many regions, the disease remains a challenge for the veterinary and public health sectors, especially in developing countries and in high-income nations with wildlife reservoirs. Current bTB control programs are mostly based on test-and-slaughter, movement restrictions, and post-mortem inspection measures. In certain settings, contact tracing and surveillance has benefited from *M. bovis* genotyping techniques. More recently, whole-genome sequencing (WGS) has become the preferential technique to inform outbreak response through contact tracing and source identification for many infectious diseases. As the cost per genome decreases, the application of WGS to bTB control programs is inevitable moving forward. However, there are technical challenges in data analyses and interpretation that hinder the implementation of *M. bovis* WGS as a molecular epidemiology tool. Therefore, the aim of this review is to describe *M. bovis* genotyping techniques and discuss current standards and challenges of the use of *M. bovis* WGS for transmission investigation, surveillance, and global lineages distribution. We compiled a series of associated research gaps to be explored with the ultimate goal of implementing *M. bovis* WGS in a standardized manner in bTB control programs.

## 1. Introduction

Tuberculosis (TB) is a transmissible disease of humans and animals accompanying societies for thousands of years [1]. Despite progress in its control and prevention, TB is a top cause of mortality by a single infectious agent in the world and has devastating effects on bovine livestock and wildlife populations. Ten million new cases and 1.2 million human deaths were reported in 2018, and the increasing incidence of multidrug resistant strains is a threat to public health [2]. In addition, bovine TB (bTB) is an OIE (World Organisation for Animal Health) notifiable disease and, of the 179 countries reporting disease status in 2015–2016, approximately 50% declared the presence of TB in animals, with higher prevalence in Africa and parts of Asia and the Americas [3]. Despite an effective global notification system, the actual impact of bTB in animals is not fairly quantified, especially in wildlife and in countries where disease control programs are not well-established [4]. TB in cattle has important socioeconomic consequences, as the loss of livestock severely affects producers in developing countries with poorly implemented disease control programs and in certain developed nations where specific wildlife reservoirs create pockets of infection [4,5,6,7,8,9,10].

bTB is also a major, but often neglected, public health concern [11]. The causative pathogen of the disease can be transmitted from cattle to humans through close contact or the consumption of unpasteurized milk [11,12]. It is estimated that zoonotic TB affects 143,000 people a year, killing approximately 12,300 individuals [2]. People with zoonotic TB face arduous challenges, as most strains of the bovine pathogen carry conferring resistance mutations to pyrazinamide [13,14,15], one of the first-line drugs in TB treatment, and a possible association with extra-pulmonary disease [16] delays diagnostics and treatment initiation [17].

The risk of zoonotic TB, economic losses in affected livestock, and benefits of a bTB-free status in international commerce, makes the eradication of bTB desirable in many places. Effective programs of bTB control and eradication are typically based on test-and-slaughter, movement restrictions, and post-mortem inspection measures [18]. When performed, active surveillance and contact investigation have played major roles in reducing or eliminating the disease, benefiting from effective bacterial genotyping systems used to guide targeted interventions [19,20,21,22]. These genotyping techniques applied for *Mycobacterium bovis*, the main causative pathogen of bTB, have been historically based on the evaluation of a limited set of genetic markers mainly through PCR-based assays [23,24,25,26,27,28,29]. Although these techniques have been useful for bTB control programs, *M. bovis* whole-genome sequencing (WGS) will likely replace some of these laborious assays as the cost per genome continuously decreases, while simultaneously allowing the investigation of outbreaks for which higher resolution is warranted [21,26,30].

Genomic approaches have been successfully applied to identify pathogens, study pathogen evolution and population structure, reconstruct transmission chains, detect sources of infection, calculate rates of geographical and temporal spread of disease, and determine antimicrobial resistance [31,32,33]. WGS has increasingly become the preferential technique for infectious disease epidemiology, moving from research settings to support public and veterinary health professionals in their decision-making process regarding treatment, outbreak response, and surveillance [21,34,35,36,37]. Accordingly, the World Health Organization (WHO) and the OIE have issued general and/or pathogen-specific technical standards for adopting WGS-based approaches in diagnostics, treatment guidance, and epidemiology studies [38,39,40,41]. More specifically for *M. tuberculosis*, the pathogen of human TB, WGS is being implemented in certain countries to direct patient treatment and improve surveillance systems [34,42]. In addition, the WHO launched a technical guide for routine genotypic drug susceptibility testing (DST) [41] to substitute traditional phenotypic assays, which will allow fast and accurate detection of resistant pathogens in the near future. As for *M. bovis*, certain developed countries have started to apply WGS in official bTB control programs over the past years [21]. Nevertheless, *M. bovis* specific guidelines for WGS data analysis are not yet available. Although it is possible that much of what is being developed for *M. tuberculosis* will be applicable to *M. bovis*, intrinsic genomic and disease dynamics differences of both pathogens will likely influence data analyses and interpretation moving forward.

Rapid, reliable, and interpretable notification of genomics-informed data from *M. bovis* outbreaks in the future is expected to improve source investigation and contact tracing [26]. By correctly identifying the source of *M. bovis* infection, as well as the transmission links that followed it, one can provide supportive evidence to delineate interventions to halt disease spread. An ideal WGS-based notification system would be able to detect *M. bovis* transmission links, store this information, and analyze and compare transmission networks in real-time and during selected time intervals. Over time disease surveillance and global dispersal of *M. bovis* lineages are also benefiting from whole-genome based data [21,43,44,45,46,47,48,49,50]. WGS has been a powerful tool to identify *M. bovis* lineages distributed worldwide [45] and also to provide the fine resolution needed to understand bTB introduction into countries, regions, and individual farms or wildlife populations over defined periods of time [21,45,48,50,51]. However, the widespread application of WGS and its resulting data faces technical challenges that need to be addressed. These challenges are dispersed from data collection to analysis and reporting to end-users, i.e., veterinarians and epidemiologists. As many stakeholders do not routinely work with WGS and phylogenetics, there is a need to analyze and present complex genomic data in a standardized, accurate, and succinct manner to inform outbreak response. Identifying and addressing challenges of *M. bovis* WGS analysis will pave the way towards the systematic application of such technology in bTB control and eradication programs. Therefore, the aim of this review is to describe *M. bovis* genotyping techniques and discuss current standards and challenges of *M. bovis* WGS data analysis and interpretation. The section on *M. bovis* WGS is focused on its applicability for pathogen transmission investigation, surveillance, and global lineages distribution, benefiting from transferable contributions of the rich literature surrounding *M. tuberculosis* WGS.

## 2. A Brief Background on MTBC Genomics

To precisely interpret genotyping and WGS data, it is necessary to understand the genetic make-up of *M. bovis*. This pathogen is part of the *Mycobacterium tuberculosis* complex (MTBC), a bacterial group composed of 11 species or ecotypes with variable host tropism and virulence [1,52]. *Mycobacterium tuberculosis* is the leading etiological agent of TB in humans, while *M. bovis* has a broader host range and is able to infect multiple host species, mainly cattle and including humans, with variable populational persistence [52]. The MTBC is a clonal group [1,53,54,55] that evolved from a common ancestor with the tuberculous *Mycobacterium canettii* thousands of years ago [56,57]. MTBC genomes are highly similar, with >99.95% identity over homologous nucleotide sequences, including the ribosomal RNA genes, while horizontal gene transfer and large recombination events are considered absent [1,54,55]. These pathogens have solely evolved through single nucleotide polymorphisms (SNPs), indels (small insertions and deletions), deletions of up to ≈26 Kb, insertion sequences (IS), and duplication of few paralogous gene families [1,54,58].

Some of these large deletions, called “regions of difference” (RD), were initially described through physical mapping and differential hybridization arrays amongst *M. tuberculosis* H37Rv, *M. bovis* BCG Pasteur, and *M. bovis* ATCC 19210 [59,60,61]. Fourteen evolutionarily stable regions of difference (RD1–14) were differentially present among these strains and ranged from 2 to 12.7 kb in size. The discovery of these RDs paved the way towards the molecular diagnosis and differentiation of MTBC species [62], and are considered the gold-standard to differentiate members of this complex. Accordingly, *M. bovis* can be accurately differentiated from other members of the MTBC by the deleted regions RD9 and RD4, and from *M. bovis* BCG by the absence of RD1^BCG^ (which is deleted in BCG strains) [62].

The bovine tubercle bacillus was officially named *M. bovis* in 1970, albeit called this way since the beginning of the 20th century [63]. The type strain was defined as *M. bovis* ATCC 19210, still referenced in the most recent Bergey’s Manual of Systematic Bacteriology [64], along with CIP 105234 and NCTC 10772. For tuberculous mycobacteria, early taxonomic classification was based on specific phenotypic traits of the isolates, such as host of origin, virulence in animal models, and biochemical tests (e.g., pyrazinamide resistance, niacin accumulation, nitrate reduction, type of respiration, colony morphology) [64]. The high genetic relatedness between *M. tuberculosis* and *M. bovis,* as well as among other species of the MTBC*,* has always instigated discussions about their taxonomic classification, frequently suggesting to compile all members of the MTBC to a single species [65,66,67,68,69]. However, the biochemical differences and epidemiologic distinctions between infections, particularly regarding the bovine and human bacilli [63], emphasized the need for differentiating these organisms at some taxonomic level, which remains to be defined (e.g., species, subspecies, variant).

The average size of a virulent *M. bovis* genome is 4.3 Mb, containing approximately 4200 genes, including a single copy of each of the ribosomal RNA genes (5S, 16S, and 23S) and 45 tRNAs. As with other *Actinobacteria* [64], its genome has a high GC content (≈65%), which implies the use of appropriate sequencing reagents for library preparation in WGS [70]. MTBC genomes, including *M. bovis*, have a substantial number of repetitive elements, constituting one of the main challenges for WGS data analyses. These include, but are not restricted to, mobile elements (e.g., insertion sequences—IS), proline-glutamate (PE) or proline-proline-glutamate (PPE) family genes, integrases, two phage sequences, a CRISPR (Clustered Regularly Interspaced Short Palindromic Repeats), and the 13E12 repeat family genes. In particular, PE-PPE gene families account for approximately 10% of MTBC genomes, and have been associated with TB pathogenesis [71]. Repetitive elements are difficult to handle in genomic studies because the majority of and most commonly used sequencing platforms generate short reads, usually ranging from 50 to 300 bp, which are often shorter than the repeats themselves [72]. Some of these repetitive regions are the basis for the traditional genotyping techniques developed over the years (see next section).

## 3. Traditional Genotyping Techniques of *M. bovis*

**Figure 1:**
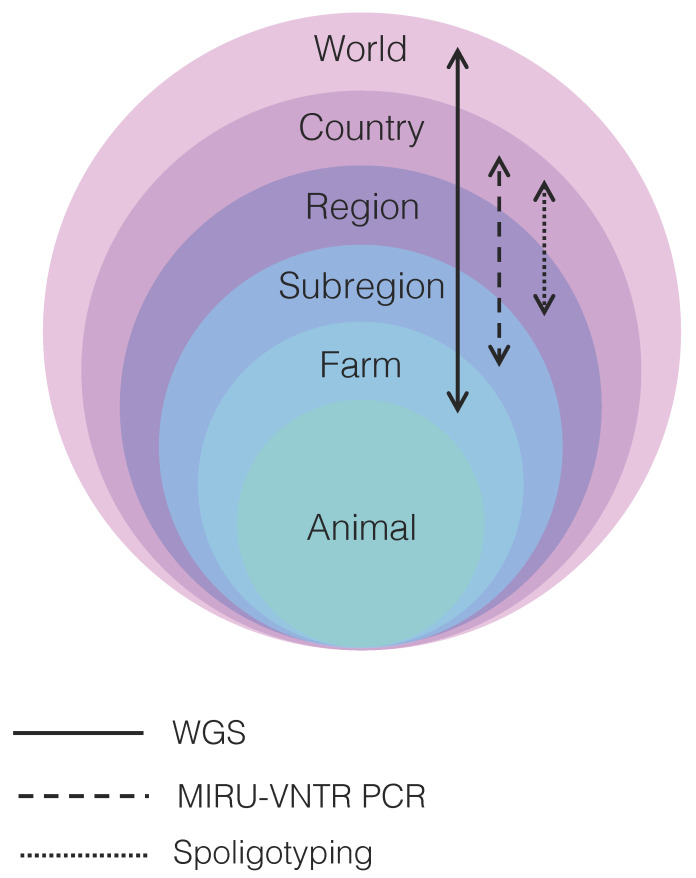
Resolution power of the main techniques used to resolve transmission clusters of *Mycobacterium bovis* depicted in relation to world, country, region, subregion, farm, and animal levels. WGS: whole-genome sequencing; MIRU-VNTR: mycobacterial interspersed repetitive unit-variable-number tandem repeat typing; PCR: polymerase chain reaction. Arrows indicate the level of resolution each technique is able to achieve. WGS provides fine resolution to discriminate between *M. bovis* strains distributed globally to the individual farm level, while MIRU-VNTR PCR and spoligotyping have more limited resolution, particularly at the individual farm level. WGS may be able to discriminate between different *M. bovis* strains infecting the same animal only if sampling is comprehensive, multiple isolate cultures are sequenced, and/or deep sequencing of the primary isolate is performed.

A number of reviews describe in detail traditional typing methods used for *M. bovis* outbreak investigations [23,24,25,26,27,28,29]. Nearly all techniques, briefly reviewed below, were first developed and applied for *M. tuberculosis* typing and later validated for *M. bovis* studies. Due to MTBC’s clonal nature, most polymorphisms in genotyping techniques originate from insertion sequences (e.g., IS*6110*) and other repeat regions (e.g., CRISPR, PE/PPE genes, PGRS genes). Evidence accumulated over the years indicates that each technique or a combination thereof presents distinct resolving power at the country, region, subregion, and farm levels [26] (Figure 1; Table S1).

### 3.1. Restriction Endonuclease Analysis and Pulsed-Field Gel Electrophoresis

In 1985, Collins and de Lisle [73,74] developed the first intraspecific typing technique of *M. bovis*, the restriction endonuclease analysis (REA). REA consists of applying three different enzymes (BstEII, PvuII, and Bcll) to digest high amounts of total DNA extracted from *M. bovis* isolates, followed by band pattern visualization on agarose gels. Despite its use in molecular epidemiology studies in certain countries at the time [75,76,77], the assay soon proved to be technically demanding, with an excessive number of small DNA fragments difficult to resolve [78] (Table S2; Figure S1). Currently, its application is mostly restricted to a reference laboratory in New Zealand, in which it was developed, and was last used for routine typing of *M. bovis* in 2011 [26].

A pulsed-field gel electrophoresis (PFGE) [79] assay was later developed for *M. tuberculosis* and other MTBC strains and resulted in improved resolution of band patterns compared to REA (i.e., larger and fewer bands). However, PFGE had two main disadvantages: first, the MTBC’s lipid-rich cell wall inhibits the action of lytic enzymes used in PFGE, preventing the proper use of the PFGE’s agarose plugs [80,81,82]; and second, comparative studies developed in later years showed that PFGE of *M. tuberculosis* strains had a lower intra-specific discriminatory power compared to other genotyping techniques that were subsequently developed [83,84]. This low discriminatory power is associated with MTBC’s clonality; the low number of polymorphic positions between strains may result in undistinguishable band patterns [85] (Figure S1). PFGE has also some intrinsic disadvantages, such as being technically demanding and time consuming (Table S2). Given the drawbacks, there are only three published reports using this technique to type *M. bovis* strains [86,87,88].

As REA and PFGE proved insufficient to discriminate *M. tuberculosis* and *M. bovis* strains, the search for polymorphic and stable genetic markers allowed the elaboration of superior typing techniques. Currently, the most widely used genetic markers are the IS*6110* (for *M. tuberculosis*), the direct repeat (DR) region (which is a mycobacterial CRISPR), the poly(GC) rich sequences (PGRS), and the variable number tandem repeats (VNTR) sequences. Each marker has its corresponding typing technique.

### 3.2. IS6110-RFLP

The 1358 bp IS*6110* is MTBC’s specific [89] and differences in its location and copy numbers is what discriminate among isolates [90,91]. This repetitive element was first described in 1990, by screening a *M. tuberculosis* cosmid library constructed in pHC79 with labelled *M. tuberculosis* total DNA [92]. Presently, the most standardized and commonly used method to detect IS*6110* in *M. tuberculosis* strains is the IS*6110*-RFLP (IS*6110*-Restriction Fragment Length Polymorphism) [93,94]. Briefly, the technique consists in extracting high amounts (2–3 µg) of total bacterial DNA, digesting it with P*vu*II endonuclease and subjecting the digested sample to standard electrophoresis on agarose gel. The agarose gel is then used to perform a Southern blot, in which the DNA fragments are transferred to a membrane, and probes complimentary to a portion of the 3′ end of the IS*6110* sequence hybridize to reveal the number of IS elements and size of generated fragments through chemiluminescence (originally radiolabeling) [89,94,95]. IS*6110*-RFLP patterns can be compared and compiled using specific computer software. *Mycobacterium tuberculosis* isolates from individuals that are part of the same transmission link often display identical IS*6110*-RFLP patterns, constituting transmission clusters. IS*6110* has also been shown to be stable over time (0.57–10.69 years to change, depending on the disease phase) [96], which means the technique can be used to study recent transmission or in long-term epidemiological studies.

A major drawback of IS*6110*-RFLP is the fact that nearly all *M. bovis* strains carries only 1–5 copies of the insertion element [91,97] and this technique has low discriminatory power in isolates containing five or less IS*6110* copies [98] (Figure S1). In other words, many *M. bovis* isolates will have the same IS*6110*-RFLP pattern, making it impossible to distinguish among them. As with REA and PFGE, IS*6110*-RFLP also requires high amounts of DNA and is labor-intensive (Table S2). For these reasons, despite being commonly used for *M. tuberculosis*, IS*6110*-RFLP was of little use for *M. bovis* genotyping.

### 3.3. PGRS-RFLP

In 1991, a Southern blot-based RFLP was developed based on the digestion of *M. tuberculosis* DNA using enzymes of four-base recognition sites [99]. One of the detected DNA fragments showing high heterogeneity among isolates was cloned and sequenced, revealing a highly repetitive sequence, identified as PGRS [100]. This fragment served as a probe to identify the presence of up to 30 PGRS copies present in MTBC genomes. Owing to the poor applicability of IS*6110* typing for *M. bovis*, PGRS-RFLP allowed significant improvement in *M. bovis* strain differentiation [98]. However, as with REA, the presence of multiple bands [101] makes it difficult to interpret [102] (Figure S1). As a Southern blot-RFLP based system, it also requires high amounts of DNA and is a laborious technique (Table S2).

### 3.4. Spoligotyping

**Figure 2:**
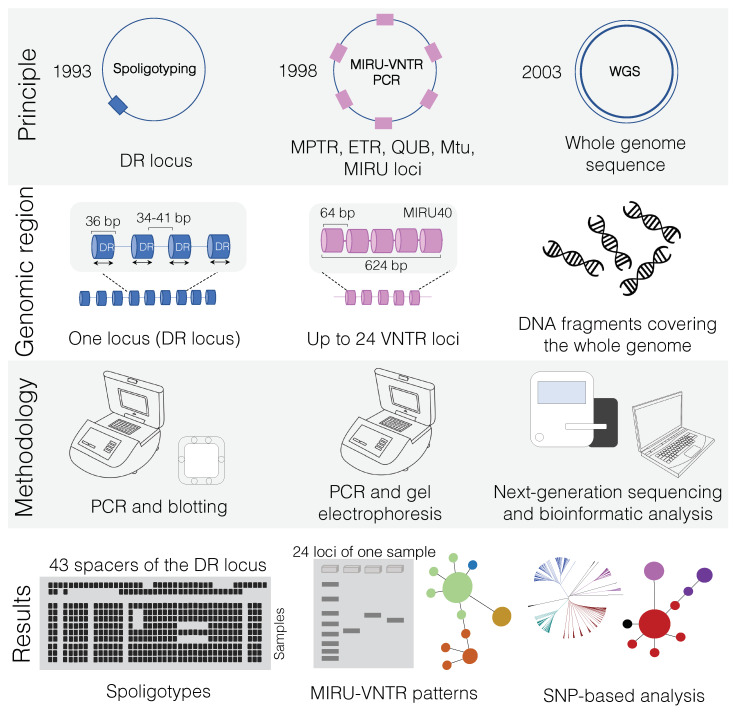
Overview of main genotyping techniques (Spoligotyping and MIRU-VNTR) and whole-genome sequencing (WGS) used for transmission cluster investigation of *Mycobacterium bovis*. In “principle”, squares denote the quantity of specific genetic markers (i.e., DR locus and VNTR) on *M. bovis* genomes. While spoligotyping is based on a unique locus, MIRU-VNTR PCR amplifies genetic targets from multiple regions of the genome (up to 24 loci). In contrast, WGS uses information from the whole-genome sequence. Dates refer to the year in which each technique was developed. In “genomic region”, the MIRU40 locus is shown as an example of one of the 24 loci that can be used in MIRU-VNTR PCR. In “results”, the spoligotyping membrane is depicted accommodating several samples simultaneously, owing to the high-throughput capability of this technique (up to 45 samples can be simultaneously analyzed). In MIRU-VNTR PCR, although many samples can be amplified at once, each sample can occupy up to 24 wells in an agarose gel, so many electrophoresis runs may be needed depending on the laboratory. MIRU-VNTR databases can subsequently be used to generate a minimum spanning tree. WGS is a high-throughput technique that will lead to single nucleotide polymorphism (SNP)-based analysis. The same generated data can also be used to detect spoligotype and MIRU-VNTR patterns (see text). WGS results can be used to evaluate transmission clusters as well as phylogenetic relationships among the sequenced genomes. WGS: whole-genome sequencing; MIRU-VNTR: mycobacterial interspersed repetitive unit-variable-number tandem repeat typing; PCR: polymerase chain reaction; DR: direct repeat.

As with many bacteria and archaea [103], MTBC organisms have a defense system against invading nucleic acids called type III-A CRISPR/Cas system. Even before much attention was given to bacterial CRISPR, this sequence in *M. tuberculosis*, known as DR locus, was described [104] and readily applied in genotyping [105]. Hermans et al. [104] originally described a genomic locus in *M. bovis* BCG containing a IS*6110* element with many 36 bp direct repeats (DRs) interspersed by spacer sequences ranging from 35 to 41 bp in size. One DR and its neighboring spacer sequence is called a “direct variable repeat” (DVR). The order of the spacers is similar among MTBC strains, but DVRs can be deleted. Therefore, the difference between two isolates is given by the variable presence of spacers in the DR region. There is only one DR locus per MTBC genome (Figure 2 and Figure S1) and up to 43 unique spacers between DRs.

The first typing method for the DR locus was called DVR-polymerase chain reaction (DVR-PCR) [105], which was later substituted by spoligotyping (spacer oligotyping technique). Spoligotyping was developed in 1997 [106] and readily utilized to evaluate *M. bovis* strains [107]. This “reverse line blot hybridization technique” is PCR-based and detects the presence of the unique spacers in an MTBC isolate in two steps. First, the spacers between DRs are amplified using PCR. A single primer set complimentary to the two extremities of the DR sequences is used, but the reverse primer is biotin labelled, resulting in the synthesis of labelled reverse strands. Individual spacers are subsequently detected by hybridization of the biotin-labelled PCR product to a nylon membrane containing covalently linked oligonucleotides corresponding to 37 spacers of *M. tuberculosis* H37Rv and six spacers of *M. bovis* BCG. A mini-blotter is used for hybridization and up to 45 isolates can be simultaneously compared [28] (Figure 2). One advantage of this technique is that it can be applied directly to DNA extracted from infected tissue samples, not requiring bacterial isolation [108]. In the case of *M. bovis*, spacers 3, 9, 16, and 39–43 are lacking, allowing for species differentiation [106].

Further improvement and automatization of the technique led to the application of microbead-based detection systems, such as Luminex platforms [109,110,111], multiplexed primer extension-based spoligotyping assay using automated matrix-assisted laser desorption ionization-time of flight mass spectrometry (MALDI-TOF MS) [112], microarray [113,114,115,116], and ligation-based amplification and melting curve analysis [117].

The evolution of spoligotype patterns is given by the loss of spacer sequences, which cannot be restored by recombination and is, therefore, fixed in that population [118]. The problem of spoligotyping is the homoplasy, i.e., unrelated lineages can present identical spoligotype patterns because the loss of spacer sequences is a common event [119]. Thus, spoligotypes are not good indicators of phylogenetic relatedness. In addition, its resolving power has been frequently shown to be lower than REA and MIRU-VNTR PCR (mycobacterial interspersed repetitive unit-variable-number tandem repeat typing, polymerase chain reaction) [26] (Figure 1; Tables S1 and S2). Despite these factors, spoligotyping remains as one of the most applied genotyping techniques in *M. bovis* studies (Table S1).

### 3.5. Variable Number Tandem Repeat (VNTR)

VNTR is a locus in which a nucleotide sequence is arranged as tandem repeats, i.e., repeats clustered together and oriented in the same direction. The size (in bp) of this locus varies according to the number of times the nucleotide sequence is repeated. Each repeat can be added or removed through recombination or replication errors, resulting in alleles with different number of repeats. VNTR are present in eukaryotes and prokaryotes, and given its variability, it has been frequently used for DNA typing [120].

Compared to the single DR locus of spoligotyping, many VNTR loci exist in MTBC (Figure 2 and Figure S1) and they are detected using PCR. The sizes of resulting PCR products correspond to the number of repeats in each locus. Initially, 11 VNTR loci (five MPTR loci, with repeats of 15 bp, and six ETR loci, with repeats of 53–75 bp) were evaluated in MTBC strains [121,122,123]. Five ETR loci (ETR-A, -B, -C, -D, -E) showed more discriminatory power among strains [124]. However, these five loci did not provide higher resolution compared to the IS*6110*-RFLP in *M. tuberculosis* strains with high IS*6110* copy numbers [121,124]. Since *M. bovis* has few IS*6110* copies, ETR loci were indeed more discriminative than IS*6110*-RFLP [121,125], but spoligotyping continued to present higher resolution [124,126]. Thus, other loci were identified and tested, such as MIRU, QUB, and Mtu, and currently a 24-loci MIRU-VNTR PCR is commonly used [127]. MIRU-VNTR can also be evaluated along with spoligotyping to infer genotyping through the online platform MIRU-VNTRplus [128], providing a standardized manner of results delivery.

Among *M. bovis* studies, different sets of VNTR loci have been applied (Table S1). Each locus and combination thereof may present better or worse discriminatory power depending on the region and sample set (Table S1). It has been suggested that each region should define the best combination of loci for its reality [129], aiming also at decreasing the cost and time spent in running different PCR assays. It has also been shown that, in certain settings, the capacity of MIRU-VNTR PCR in detecting transmission clusters may be dependable on the *M. tuberculosis* lineage [130,131]. For instance, it has been described that standard 24-loci MIRU-VNTR PCR has low resolution power to precisely discriminate closely related isolates of the lineage 2, Beijing of *M. tuberculosis* [131]. It is unknown if this is also the case for *M. bovis* lineages or clonal complexes.

## 4. The Dawn of a New Era: WGS to Understand *M. bovis* Epidemiology and Ecology

The first complete genome sequence of *M. bovis* to become available originated from a strain denominated AF2122/97 isolated from a cow in England [132,133]. As this was the first *M. bovis* genome available, *M. bovis* AF2122/97 is now considered the reference genome of *M. bovis* in GenBank, as genomes of *M. bovis* type strains have never been sequenced. By December 2019, only 74 virulent *M. bovis* genomes (i.e., not BCG) are deposited as complete or draft forms in NCBI, compared to 6522 *M. tuberculosis* genomes. In SRA (Sequence Read Archive), the database for depositing raw reads, the number of sequenced *M. bovis* is in the thousands. The disparity in numbers between assembled complete or draft genomes and raw reads highlights that the majority of developed studies are based on SNP and/or indel detection using reads.

### 4.1. Current WGS Workflow

#### Overview

**Figure 3:**
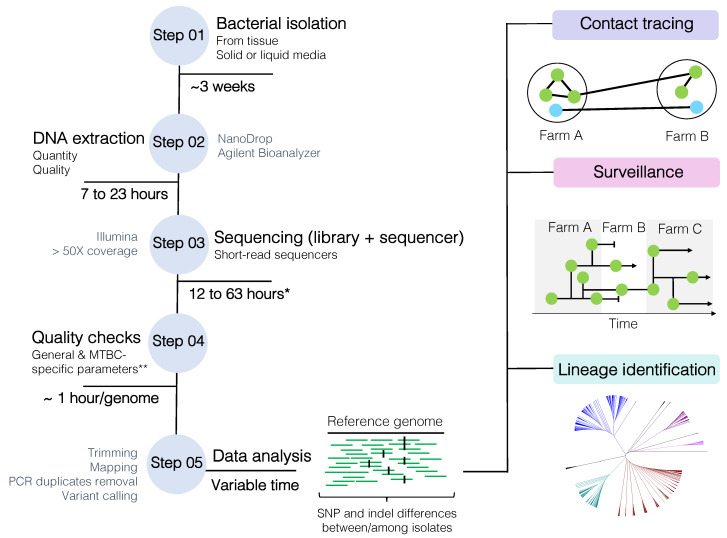
*Mycobacterium bovis* whole-genome sequencing (WGS) workflow from bacterial isolation to data analysis. SNP: single nucleotide polymorphism. * Time is highly dependable on library kit and sequencing protocol. ** MTBC-specific and general parameters are described in detail in the text, but overall this includes FastQC parameters, minimum established sequencing coverage, contaminating reads, species confirmation, mixed-strain evaluation (depending on the purpose of the analysis), and homogeneous sequencing coverage (after reference genome mapping). MTBC: *Mycobacterium tuberculosis* complex.

The current WGS workflow (Figure 3) begins with the isolation of *M. bovis* from de-contaminated tissue samples on solid (e.g., Stonebrink, 7H11-OADC) or liquid media (e.g., 7H9-OADC, MGIT—mycobacterial growth indicator tube), followed by the extraction of its DNA, library preparation, and WGS using short-read technologies (e.g., Illumina platforms). Special attention must be given to the quality of extracted DNA and the use of library kits that can accommodate high-GC content bacteria [70]. DNA extraction of mycobacteria is not trivial; the lipid-rich cell wall interferes with yield and DNA purity, which may affect library construction [70]. An optimized extraction protocol of non-tuberculous mycobacteria for long-read sequencing has been recently proposed [134]. Once DNA is successfully extracted and sequenced, generated reads need to undergo quality checks and are processed in specific data pipelines tailored to each need. For epidemiology purposes, WGS can be used to assess the genetic relatedness among isolates to address transmission investigation, surveillance, and/or lineage identification (Figure 3). Current methodologies are typically based on the identification of SNP and indel differences between or among isolates. Basically, the greater the SNP and indel difference between two isolates, the lower the probability they are related to each other. SNPs and indels are ultimately identified by mapping the quality-checked reads to a reference genome and calling the variants.

## 5. Data Analyses Pipeline

### 5.1. Quality Assessment of Entry Data

The quality of WGS reads can dramatically impact the study outcome. Therefore, quality assessment is considered the first step in data analyses. Once laboratory-specific [135] and standard quality controls associated with the sequencer run are evaluated and errors originating from the sequencer itself are ruled out, generated FASTQ files normally undergo general and mycobacteria-specific quality assessments and processing. Accordingly, following adaptors removal with appropriate software [136,137,138,139], an overall quality evaluation of reads is typically performed using FastQC [140] or similar software [139,141,142,143]. Quality parameters are frequently evaluated rather manually, by analyzing the QC report of each FASTQ file or by using software that compile multiple-sample QC reports [141,142]. Nevertheless, these are important measures to ensure that high-quality sequencing data are used in downstream analyses. Based on this QC evaluation, FASTQ files are usually processed to remove low quality data.

Parameters such as anomalous GC content, duplicated sequences, per base and per sequence qualities, per base N content, sequence length distribution, among others can be addressed according to pre-established and/or default thresholds. The detection of anomalous GC content may indicate possible sample contamination, as any peak differing from the high value of mycobacteria (≈65%) are not expected. A high level of sequence duplication indicates errors or enrichment bias related to PCR amplification and sequencing that are not expected to occur in WGS [140]. Duplicated sequences (e.g., PCR duplicates) are normally removed downstream in the pipeline, after read mapping, using appropriate software [144]. In addition, reads are often trimmed and filtered out according to quality, with user-specified or default thresholds. Protocols of trimming with different stringency levels have been tested for eukaryotes, showing that variations of parameters may significantly affect end-results [145,146]. Basic FASTQ processing (e.g., adaptor removal, read trimming, read filtering, removal of duplicates, among others) can be performed by using a combination of different software or by using all-in-one tools [143]. Finally, QC results should be evaluated before and after file processing to guarantee that minimum quality standards have been reached with appropriate read length distribution.

Unfortunately, sequencing files can often contain contaminating reads, i.e., reads not originating from the target genome [147,148,149,150]. These contaminants may or may not result in discrepant GC content peaks. Their presence is sometimes inevitable and challenges for eliminating contaminant reads have been addressed previously [34]. If not evaluated beforehand, their presence may be detected only at read mapping or genome assembly, or go undetected and result in false positive or negative SNPs [151]. One way to check for contaminants is to use FastQ Screen [152] or Kraken [153], and if desired, filter out unwanted reads following a pre-established threshold of sample contamination acceptance [152]. One study using Kraken defined a threshold of at least 90% of the reads taxonomically assigned to MTBC for the sample to be included in the analyses [154]. However, because MTBC genomes are highly similar, it is difficult to control for cross-contamination when sequencing several MTBC isolates at once [155]. Heterozygous sites may occur, and the sample falsely considered a mixed-infection.

There are three mycobacteria-specific sequencing quality criteria that can be evaluated: (i) homogenous sequencing coverage; (ii) RD identification; and (iii) within-host genetic diversity. One of the advantages of MTBC clonality is that read-mapping coverage to a reference genome can be utilized as measure of homogeneous sequencing coverage of the target genome. When mapping high-quality reads to a MTBC genome, a high mapping coverage (>95%) of the reference genome is expected [156,157,158,159]. Percentage cut-offs may be established [45,160], because substantially low percentages are likely to indicate that the target genomes were not evenly sequenced. In addition, the presence of species-specific RDs in the target genome must be assessed. In our laboratory, we have identified a number of MTBC genomes deposited in public databases with mistakenly-assigned MTBC species [45,161]. As MTBC members have high genomic and phenotypic similarity, errors in species identification may occur. Therefore, even if the bacterial isolate was obtained from cattle tissue, *M. bovis* specific RD patterns should be confirmed. This confirmation can be performed using reads, by checking RD regions through reference genome-mapping [45], or by running the automated software RD analyzer [162].

Another major challenge of SNP-based approaches for MTBC WGS analysis is within-host genetic diversity [163]. The amount of MTBC genetic diversity an individual carry depends on the time between infection and development of active disease (within-host evolution, i.e., microevolution) and/or the number of strains this individual was exposed at single or multiple infection events through life (mixed-infection) [163]. Microevolution occurs during long-term co-existence between pathogen and host and is characterized by a single infection event leading to bacterial mutations over time. On the other hand, mixed-infection occurs when the individual is exposed to a single or repeated infection events through life of different strains, and is thus carrying distinct strains of MTBC [163]. If DNA is extracted from the primary isolate without bacterial propagation from a single, de-clumped colony, there may be simultaneous sequencing of more than one strain in a sample. Thus, when mapping reads to the reference genome and calling variants, heterozygous sites may arise. More details regarding this issue are given in the following sections.

### 5.2. Choice of Reference Genome for Read Mapping

A closed, complete genome must be chosen as reference for read mapping and variant calling. The choice of reference genome can dramatically alter the end-results [164,165] and it is still a controversial matter [34]. Lack of standardization of reference genomes halts comparisons between pipelines and laboratories. Ideally, the reference genome must have all DNA segments present in the bacterial population under study. If the reference genome has deleted regions compared to the genomes being tested, genetic diversity may be missed. The evolutionary distance between the microbial genomes under study and the reference genome should also be taken into account [165,166]. For instance, if *M. tuberculosis* genomes are used as reference for *M. bovis* studies, the number of detected SNPs increases dramatically [47,167], which may lead to errors in read mapping and variant calling [165], substantially increasing computer usage and time.

WGS-based studies of *M. bovis* often use as reference the genome of *M. bovis* AF2122/97 (Table S3). One recent study has proposed the use of an outbreak-matched *M. bovis* genome as reference in France [168]. Studies of *M. tuberculosis* have used the *M. tuberculosis* H37Rv genome, lineage- or outbreak-matched genomes, or an inferred ancestral MTBC genome, which have been reviewed elsewhere [34]. The use of a MTBC pan-genome as reference, i.e., a gene pool representing the whole diversity of MTBC genes, has also been suggested, but never evaluated [34]. Intergenic regions, however, should not be neglected in future technical validations of these approaches. Recently, a computational pan-genome of *M. tuberculosis* (in this case, a dataset of whole genome sequences, and not simply core and accessory genes) with 5,205,216 bp obtained from 146 *M. tuberculosis* genomes has been proposed as a reference genome for this species [169]. Considering that a *M. bovis* transmission cluster may be defined or ruled out by just few SNPs (see following sections), more comprehensive studies on the effect of the reference genome on SNP identification should be performed.

### 5.3. Reads Mapping and Variant Calling

Bacterial SNPs and indels, as well as structural variants (SVs—indels, duplications, inversions, and translocations >50 bp), can be identified through *de novo* genome assembly followed by comparison against a reference genome, or by mapping reads to a reference genome [170]. When using assembled genomes, failure to properly identify variant calls can occur due to assembly errors or misidentification of indels [170]. It is more appropriate and faster to use the complete information provided by the reads than relying on assemblers and consensus base callers [171] to detect variants. Thus, mapping reads to a reference genome is the preferred first step to detect high quality variants. Numerous short-read aligners have been developed (to cite a few [171,172,173,174,175,176,177,178,179,180,181]) and additional information about mapping principles have been reviewed [179].

Different approaches of read mapping and variant calling are described in *M. bovis* studies (Table S3). Among the most widely used short-read mapping tools are Bowtie/Bowtie2 [172] and BWA/BWA-SW [174]. Output alignment files are subsequently processed to call and generate a list of high-quality variants using tools available from toolkits or pipelines such as VarScan2 [182], SAMtools [173,183], and GATK (Genome Analysis Toolkit) [184,185]. PCR duplicates are frequently removed after read mapping with Picard (MarkDuplicates; https://broadinstitute.github.io/picard/) or SAMTools (rmdup) [173,183]; but the actual necessity for this step has not been systematically evaluated using MTBC genomes. VarScan2 combines a heuristic method coupled with statistical algorithm to detect mutations from read mapping, and integrates identification of SNPs, indels, or both (mpileup2snp, mpileup2indel, mpileup2cns, respectively). On the other hand, SAMtools and GATK are probabilistic methods, implementing Bayesian statistics. SAMtools contemplate the bcftools to call variants [173], while GATK version 4 uses HaplotypeCaller [184,185]. Unfortunately, variant callers have been mostly benchmarked with human genomes, which may lead to the report of false variants when analyzing microbial genomes [170]. More recent studies showed marked result differences among pipelines for variant detection in WGS studies of *M. tuberculosis* [160,165] and other bacteria [166,186].

One of the reasons for these discrepancies is that MTBC studies vary widely on the parameters adopted to map reads and call high-quality variants; no standards have been determined. The choice of parameters greatly influences variant detection (e.g., base call and mapping quality scores, tail distance, presence of variants on both strands for paired-end reads, read depth, minimum allele frequency, maximum number of SNP calls within 10–12 bp, local assembly or realignment around indels, and strand bias) [170]. Sequencing coverage, PCR duplicates, mapping artefacts around indels, SVs and repetitive or duplicated regions may also result in false positive (FP) and/or false negative (FN) calls [170]. As part of MTBC-specific measures, it is common practice to exclude SNPs and indels associated with repetitive DNA, such as PE/PPE family genes, phage genes, repetitive family 13E12 genes, transposases, and integrases, following their identification through annotation or genomic position, or by excluding selected genes from the reference genome [165,187]. However, comprehensive evaluation of the true probability of these calls being FP or FN are lacking. By using read simulation and comparison to long-read sequencing, a recent study has shown that SNPs detected in PE/PPE regions were highly unlikely to be FP calls when using BWA read mapping and Pilon variant caller [188] (which is a microbial variant caller). In contrast, another study has shown that both FP and FN calls are disproportionately present in PE/PPE regions in a multi-variant caller comparison [165]. This contradiction highlights the need for additional studies. Thus, significant challenges remain to be overcome in order to define the best parameters to call variants and how to handle low-quality variant calls.

### 5.4. Within-Host Genetic Diversity and Its Impact on Variant Calling

Two major technical challenges arise in variant calling when there is within-host genetic diversity: establishment of minimum allele frequency to identify a site as heterozygous, and the minimum number of heterozygous variants in a sample to be considered a “mixed-sample” condition. Importantly, the ability to uncover these parameters in *M. tuberculosis* studies is also directly affected by sequencing coverage [189]. The minimum allele frequency is a fixed threshold (≈75% to 95%) for the proportion of reads supporting a particular variant call. Sites with the percentage of reads falling below this threshold are considered heterozygous and hence used to support a mixed-infection. Unfortunately, there is no consensus on the percentage level that should be used.

Once heterozygous sites have been identified, two strategies are commonly applied to determine a mixed-sample condition: a cut-off proportion of heterozygous sites to total variants, or a minimum total number of heterozygous sites [21,45,51,190,191,192,193,194,195]. Certainly, the percentage threshold depends on the choice of the reference genome, e.g., *M. bovis* AF2122/97, *M. tuberculosis* H37Rv, or reconstructed MTBC ancestor. Currently, there are no established criteria or thresholds of what can be considered mixed-sample and what is variant calling error for *M. bovis*, especially in light of repetitive genomic regions and different parameters set for variant calling. Once a *M. bovis* mixed-sample is detected, researchers have either removed the sample from downstream analysis [21,45,51,192,194], consider these heterozygous sites in the context of the contact chain being analyzed to resolve transmission networks [21,194,196,197,198,199], or excluded heterozygous sites from downstream analysis [43,193,200,201,202].

### 5.5. Within-Host Genetic Diversity and Its Impact on Transmission Detection

Individual animals or humans carrying MTBC isolates with distinct SNP profiles have been described [21,48,51,156,163,194,195,196,197,198,203,204,205,206,207,208]. As explained above, such conditions occur when there is microevolution and/or mixed-infection. Both concepts, the manner in which within-host genetic diversity is detected, and their application to the definition of transmission clusters have been initially defined with *M. tuberculosis* and later applied to *M. bovis* WGS studies [21,45,47,51,194,195]. Owing to the low substitution rate of MTBC [46,190,196,202,209,210], the number of acquired SNPs by *M. tuberculosis* strains under a microevolution process (within-host evolution) has been estimated to be very low, usually in single digits [29,163,190,196,197,209,211]. On the other hand, mixed-infection is defined when two or more *M. tuberculosis* isolates obtained from an individual differ by a great number of SNPs [29,163,196].

**Figure 4:**
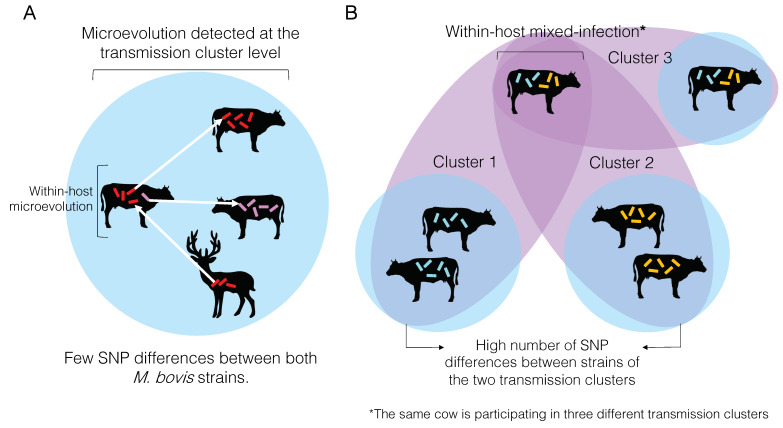
Overview of microevolution and mixed-infection conditions and its relationship to and influence on the detection transmission clusters of bovine tuberculosis. (**A**) Microevolution condition. Microevolution is normally determined when two *Mycobacterum bovis* isolates obtained from the same host differ by a small number of SNPs (usually between 0 and 12 SNPs; see text). The same SNP threshold is used to define a transmission cluster, when two *M. bovis* isolates obtained from different hosts also differ by the same number of SNPs. (**B**) Mixed-infection condition. Mixed-infection is defined when two isolates obtained from the same host differ by a great number of SNPs (usually > 12 SNPs; see text). When a great SNP distance is found between two *M. bovis* isolates from different hosts, these animals are not considered part of the same transmission cluster. However, if an animal is infected with two strains differing by a great number of SNPs (i.e., mixed-infection), it may be identified as participating in two different transmission clusters (cluster 1 and cluster 2). If the within-host genomic diversity is not entirely captured, one of the transmission clusters may be missed. This animal with a mixed-infection may also transmit both strains to another animal (cluster 3), and if the diversity is entirely captured, both animals will be considered as part of the same cluster.

Microevolution can be detected at the individual or transmission cluster levels (Figure 4A). Very often, microevolution is only detected at the latter, because the whole extent of within-host genetic diversity is frequently missed due to insufficient individual sampling [163]. When these low SNP distances are inferred among individual samples in a cluster, they are used to define them as part of the same transmission cluster. In other words, the same *M. bovis* strain was transmitted from one animal to the other, and the amount of genetic changes accumulated is zero or just a reflection of within-host evolution (i.e., microevolution) represented by very few SNPs. Contrastingly, if the number of SNPs between two individual samples is too high, they are not considered part of the same transmission cluster (Figure 4B).

At the transmission cluster level, if this within-host genetic diversity is captured with adequate sampling, an individual may be considered part of two transmission clusters, representing different infection events that occurred over time (Figure 4B). More likely the within-host genetic diversity is not entirely captured, and pathogen transmission between or among individuals may be mistakenly discarded. In other words, if a cow is infected with two distinct strains of *M. bovis* differing by a great number of SNPs*,* and only one is sequenced but both are transmitted to other cows, one of the animals in the transmission chain will go undetected as part of that cluster. It is important to highlight that an individual may get re-infected with the same strain, which would be impossible to distinguish using current analytical methods. The actual impact of within-host genetic diversity on the transmission dynamics and pathogenesis of *M. bovis* remains to be comprehensively studied.

### 5.6. Where to Go after Detection of Variants?

#### 5.6.1. SNP-Counting Method

Few approaches have been used to interpret variant calling data in *M. tuberculosis* and *M. bovis* studies. The simplest one is to use the absolute number of detected SNPs (indels are excluded) to infer relatedness based on predefined thresholds. This methodology has been often employed in *M. bovis* studies [21,47,51,210,212] (Table S3). Based on solid epidemiological links, SNP thresholds have been established to distinguish within-host microevolution from mixed-infection of *M. tuberculosis* [190,193,197,209]. Consequently, the same SNP thresholds were also applied to distinguish isolates belonging to a transmission cluster or not, helping define a transmission chain of *M. tuberculosis* or *M. bovis* and differentiate relapse from re-infection in *M. tuberculosis* infections [21,47,51,163,190,193,196,197,199,209,213,214,215,216,217] (Table S3). One of the first studies to define SNP thresholds evaluated *M. tuberculosis* isolates obtained from chronically infected patients, epidemiologically linked patients, and outbreaks with confirmed transmission chain observed in the UK (a low-burden country) from 1994 to 2011 [196]. A maximum of five SNPs was defined as the limit to infer a transmission cluster or microevolution. Similar thresholds have been confirmed in later studies performed at low and high burden settings [190,197,209,211], and is commonly accepted that five SNPs can be used as a stringent threshold and 10 or 12 as a more relaxed threshold [29,163]. Nevertheless, SNP thresholds described in the literature of *M. tuberculosis* vary [163]. It is known that these thresholds may be influenced by variant calling protocols, culture or sampling, read depth, and epidemiological links used to first define them, which makes them unlikely to be adequately transferred between settings and studies [163]. Thresholds have never been determined for *M. bovis*, which is likely subjected to different evolutionary pressures compared to *M. tuberculosis*. Moreover, owing to the possibility of false positives, indels are usually excluded from the analysis; just few studies of *M. tuberculosis* WGS have used this information to better resolve clusters [209,218].

Established SNP thresholds defining recent transmission events were calculated according to the evolutionary rate of *M. tuberculosis*, reported as 0.3–0.5 SNP per genome per year [190,196,209]. It is unknown if the same rate applies to *M. bovis*. Estimated substitution rates of *M. bovis* range from 0.15 to 0.53 substitutions per genome per year [46,202,210]. However, these studies either examined a limited number of isolates [210] or geographically restricted samples [46,202]. The correct estimation of *M. bovis* substitution rates has significant implications for the definition of the amount of genetic changes needed to define a transmission cluster, and for the temporal resolution WGS can provide to study disease dynamics in bTB [210]. Although it is possible that *M. bovis*-derived SNP thresholds are not very different from *M. tuberculosis*, the paucity of knowledge regarding *M. bovis* evolution and DNA repair mechanisms implies that more in-depth evaluation should be conducted. It is unknown, for instance, if the phenotype of broad host tropism [52] influences replication and substitutions rates of *M. bovis* over time.

#### 5.6.2. Whole-Genome Based Multi-Locus Sequencing Typing

Traditional MLST (multi-locus sequencing typing) is based on the identification of mutations in a pre-established, limited number of bacterial genes. In order to incorporate the whole gene repertoire of a bacterial species and WGS technology, cgMLST or pgMLST (core genome or pan-genome MLST) schemes based on MTBC core or pan-genome genes (core genes plus some accessory genes), respectively, have been applied [219,220,221,222]. Briefly, the obtained list of SNPs (indels are normally excluded) is translated into a standardizable allele numbering system. SNPs are identified in a pre-defined allele dataset of selected MTBC species; any particular gene identified with a SNP is giving a number. Each sample is then given a sequence type (ST) determined by a combination of allele numbers. In other words, these schemes are based on the concept of allelic variation. STs generated from the bacterial population under study can then be used to generate minimum spanning trees to define transmission clusters [219,220,221,222]. One great advantage of these methods is the possibility of generating a nomenclature that can be readily compared between laboratories, which is vastly appreciated for disease control and eradication programs. However, by using these approaches, information from intergenic regions may not taken into account, unless intergenic loci are added to the initial reference dataset. In addition, there may be variation in the gene pool or gene annotation inconsistencies among different strains of *M. bovis* and MTBC [156,161,223,224] that may lead to errors when initially defining the gene repertoire to serve as alleles. This approach has been independently applied in *M. bovis* isolates from a Brazilian State approaching bTB eradication status, revealing recent transmission between farms and multiple *M. bovis* introductions within the same farm [225].

#### 5.6.3. Phylogenetic Approaches

Most *M. bovis* WGS studies use phylogenetic methods to define potential clusters of pathogen transmission, to evaluate populational structure of *M. bovis*, and/or for surveillance purposes [21,43,45,46,47,48,49,50,51,161,167,194,202,210,212,217,226,227,228,229] (Table S3). In general, phylogenetic trees are constructed from alignments (i.e., matrices) of concatenated SNPs identified in each *M. bovis* genome under study. These trees are generated using different algorithms, such as maximum likelihood, maximum parsimony, neighbor-joining, or Bayesian inference. This approach provides clusters of associated *M. bovis* isolates, but additional analyses are normally performed to ascertain a transmission chain [21,44,47,50,51,202,210,212]. In phylogenetic trees, transmission pairs do not always appear phylogenetically related or associated; phylogenetic trees are not a complete substitute for a transmission network [163,230,231]. Nevertheless, Bayesian inference schemes have also been used to estimate temporal scales of bTB outbreaks by dating ancestries of the bacterial population under study [43,46,49]. When used to study *M. bovis* populational structure or evolutionary dynamics in countries or globally, phylogenetic reconstruction has always been the preferred method [21,45,46,48,50,167,194,226,227,228,229] (Table S3). However, it is important to understand that only core SNPs will be considered. All indels and variant sites that are not present in all strains are excluded from the analysis.

## 6. Errors Arising from Indels and Repetitive Regions

Genomic regions containing homopolymers or tandem repeats can lead to false reports of indels and/or SNPs due to sequencing errors or inaccurate read mapping. In addition, small and large indels are difficult to be accurately detected [154,170,232]. Therefore, current pipelines to infer *M. tuberculosis* or *M. bovis* transmission normally exclude indels or variants detected in repetitive, duplicated, and/or low-complexity regions [21,34]. Repetitive regions and duplicated genes are likely subjected to distinct evolutionary rate [233]. Thus, with the advent of more accurate variant callers and parameters, as well as long-read sequencing, the inclusion of such sites may provide further resolution for outbreaks as well as changes to the current SNP thresholds for definition of a transmission cluster in the future. Long-read sequencing technologies are powerful and promising tools that can uniquely identify the genomic origin of the read, helping resolve repeat regions, and determining large deletions or rearrangements [234]. However, a major drawback of such technologies is the low base calling accuracy when compared to short read technologies, which is detrimental for variant detection [234,235]. Hybrid systems, including the association of long- and short-read data, have been proposed to correct base calling errors [234,236]. In the future, it is expected that an increased accuracy in base calling of long-read technologies will revolutionize genome sequencing. Advantages and disadvantages of long-read sequencing compared to short-read sequencing have been recently reviewed elsewhere [234].

## 7. Software to Define Spoligotyping and MIRU-VNTR Profiles Using WGS Data

WGS does not eliminate the identification and reporting of spoligotypes and MIRU-VNTR patterns of samples under study. SpolPred [237] and SpoTyping [238] are two software developed to detect spoligotypes from short-read sequences in FASTQ format. SpoTyping also accepts assembled contigs in FASTA format as input and is reported to be 20–40 times faster than SpolPred [238]. Both software have reported identical spoligotypes in a dataset tested [238]. More recently, a methodology to reconstruct the whole CRISPR locus of MTBC strains have been proposed [239] and is awaiting further investigation for its applicability as a typing tool.

In contrast to spoligotyping, which is based on a single locus, the identification of MIRU-VNTR profiles using WGS data from short-read sequencing has been more challenging. An algorithm to assign 24-loci MIRU-VNTR profiles to isolates using draft and complete genomes have been described [240], provided that genomes meet a minimum-quality assembly. More recently, a software that uses long-read sequences obtained using Pacific Biosciences and Oxford Nanopore Technologies as input data has been developed [241], aiming to overcome the difficulties encountered with the long repeats of the MIRU-VNTR loci that may not be resolved with short-read sequencing.

## 8. Association of WGS with Epidemiological Data for Transmission Inference

Interpretation of genotyping and WGS data is challenging because the sampling of the population of interest is often partial and/or biased, and there is a variable interval between time of infection (i.e., when the transmission occurred) and time of sample collection [43,210]. In addition, transmission routes and intervals may be uncertain due to the slow evolving rates of the MTBC [46,190,196,202,209,210] and possible differences in substitution and replication rates between active replication state and latent state [43,210,242]. To circumvent some of these issues, the population of interest should be sampled in a manner that the epidemiological processes are captured [46,210].

Sole genetic data may not be sufficient to detect transmission in human or bovine TB outbreaks [26,163]. Identified transmission networks based solely on genetic data can be different from the network of actual transmission events if detailed field investigations are not performed [26,163]. In particular, highly clustered transmission networks can introduce uncertainty to the evaluation of transmission dynamics, especially when lower resolution genotyping is applied (e.g., MIRU-VNTR PCR and spoligotyping). Challenges associated with clustered networks have been reviewed elsewhere [26], but in general, clustering adds uncertainty to the identification of infection source and transmission patterns. To provide better resolution, the genetic data must be dense (i.e., well sampled) and complemented by good quality, collected epidemiological and demographic parameters. Accordingly, the association between WGS and network data have been elegantly applied to investigate bTB outbreaks at the local level [51,210]. The association between network, spatial-temporal mathematical models and WGS is the ideal situation to correctly describe the transmission dynamics of a particular outbreak [26,51,210] and propose targeted interventions. These are very powerful approaches to delineate disease control strategies in the long-term, particularly in a multi-host system; however, such refined analyses may not be easy to implement in bTB control programs requiring real-time transmission investigation.

## 9. Data Reporting in WGS Pipelines

**Figure 5:**
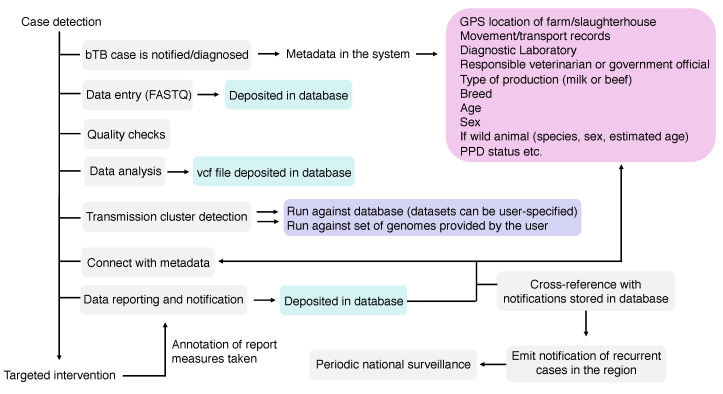
Components of a *Mycobacterium bovis* whole-genome sequencing (WGS) pipeline. In grey: components of the pipeline; in green: files that will compose the sequence and analysis databases (raw reads—FASTQ—and *vcf* file); in pink: metadata, which can also compose a metadata database linked to each FASTQ and *vcf* file; in purple: the possibilities of genome comparisons: (i) a user can choose to compare the genome against all genomes of the database or against a subset of genomes composing the database; or (ii) a user can input several genomes that can be compared against each other or with other genomes deposited in the database. An ideal pipeline would also allow periodic national surveillance reports, emitting alerts of newly detected clusters or outbreaks in certain regions that warrant further attention according to user-specified thresholds.

Once a transmission cluster, an infection source or a single infected-case or farm has been detected using WGS, such information needs to be communicated to end-users, e.g., veterinarians, epidemiologists, program officials, among others. Preferentially, reporting must be standardized and comparable among different veterinary services, connecting federal, state or province, and local stakeholders. Unfortunately, no standards exist on how these WGS reports must be, while capacity building is expected to play a crucial role in guaranteeing correct interpretation of the results. In other words, the improvement and acquirement of skills, knowledge, equipment, and general resources by personnel involved are vital for success of WGS-based programs. As bTB is an OIE-notifiable disease, *M. bovis* WGS-based surveillance systems can greatly benefit from general, robust disease reporting systems already in place in many countries [243]. An ideal system would be able to register: (i) an outbreak with genome-based transmission links that were detected using standardized data generation and bioinformatics pipelines, and (ii) individual cases or farms reporting *M. bovis* genomes that can be compared with a comprehensive database for the prospective identification of transmission links in a disease surveillance context, using the same standardized pipeline (Figure 5). A standardized bioinformatics pipeline, publicly available, has been developed by the National Veterinary Services Laboratories (NVSL) of the US Department of Agriculture (USDA), which implemented the use of *M. bovis* WGS in its official bTB program in 2013 [21]. Other pipelines (for detection of antibiotic resistance, strain typing, and/or transmission detection) have also been reported for *M. tuberculosis* and reviewed elsewhere [34]. Increasing efforts must be made to provide standardized end-to-end processes that are affordable and easily managed by non-experts.

An important example of a standardized laboratory network for WGS reporting is GenomeTrakr [37] (US Food and Drug Administration), used for foodborne pathogens. Others exist for viral pathogens [244,245]. Reflecting GenomeTrakr structure, an effective integration between veterinary, public health, university, and industry laboratories would be of utmost interest to report *M. bovis* WGS data as part of national control programs. These laboratories can undergo proficiency tests to ensure quality control and standardization in generating and depositing data to a common database [246]. Once sequencing data is deposited in public databases, further comparison and identification can be fast and efficient, provided there is an effective bioinformatic pipeline established.

Report guidelines for animal health surveillance (AHSURED) were recently proposed, aiming at a systematic description of the means by which the output of surveillance has been generated for a particular disease [247]. Through a survey of experienced professionals working in animal surveillance for State Authorities, a consolidated checklist of items to be reported was generated. Although these guidelines are not specific to any bacterial typing technique or transmission cluster identification method, its applicability using WGS data remains to be tested. Other initiatives aimed at harmonizing the documentation of disease surveillance and reporting include the SANTERO (http://santero.fp7-risksur.eu/), HOTLINE (https://www.thehotlineproject.org/), and RISKSUR (http://www.fp7-risksur.eu/) projects. Guidelines for reporting cohort, case-control, and cross-sectional studies of veterinary diseases have also been proposed (STROBE-Vet Statement; https://meridian.cvm.iastate.edu/strobe). The inclusion of pathogen WGS for infection source identification and contact tracing in these projects has never been evaluated.

## 10. Resolution Power of WGS and Genotyping Techniques

Spoligotyping and MIRU-VNTR PCR have been often applied to resolve local clustering on larger scales [26] (Table S1). However, their power to discriminate within-cluster events or at the farm-to-farm scale is rather limited [26,29,202] (Figure 1). In such instances, WGS may provide the resolution to finely resolve transmission patterns happening at the individual herd level, in clusters of small spatial extent [48,210], or in countries where bTB prevalence is almost null and re-introduction outbreaks occur due to a single-sourced *M. bovis* strain [43]. Accordingly, many studies show that WGS is useful to differentiate *M. tuberculosis* strains with identical MIRU-VNTR genotypes, proving superior resolution [29,197,209,248,249]. Frequently, traditional typing methods of *M. bovis* depict the same or few genotypes distributed over relatively large local areas or encompassing a great proportion of the tested isolates under study [29,48,210,250,251,252]. Such lack of resolution is troublesome for the detection of *M. bovis* transmission between farms or between cattle and wildlife, especially in regions approaching free-status, with low bTB prevalence. WGS may provide an opportunity to solve this problem.

On the other hand, WGS studies evaluating transmission of *M. bovis* in high-burden countries or regions with high *M. bovis* genetic diversity are lacking. Sometimes, the genetic diversity given by MIRU-VNTR and spoligotyping is so high in the region and/or in the sample set being tested that an infection source cannot be accurately identified [199,253]. The applicability of *M. bovis* WGS in these instances remains to be elucidated.

The rate of genetic variation of a pathogen has implications for the scale at which the epidemiological events can be resolved using DNA typing data [254]. Accordingly, the use of WGS has been particularly advantageous to trace RNA virus outbreaks, owing to their high substitution rate. However, MTBC has a much lower evolutionary rate compared to these pathogens. As such, the resolution power of WGS for MTBC at the animal-to-animal or human-to-human level may be poor depending on the scenario [196,202,209,210,255]. In other words, zero or only very few SNPs between or among MTBC isolates are detected, leading to a failure in describing transmission links carrying meaningful information for prospective interventions. This is not a restraint of the WGS technology per se, yet a consequence of the low mutation rate of MTBC when compared to fast evolving pathogens, such as viruses. Regardless of this limitation, for both *M. tuberculosis* and *M. bovis*, it has been concluded that the epidemiology of outbreaks can greatly benefit from WGS data, providing better resolution than any other genotyping technique [26,34,202,249,256,257].

In a bacterial genome, repeat regions exhibit faster evolutionary rates compared to non-repeat regions [233]. MIRU-VNTR and spoligotype genomic regions have been successfully applied for genotyping because these are rapidly evolving regions of repetitive DNA. As explained above, the loss and gain of fragments within these regions drive the identification of genotyping patterns. Therefore, the genetic variation given by MIRU-VNTR PCR and spoligotyping is not depicted in current whole-genome data interpretation, which is based on SNP divergences. It also means that WGS is presently based on signals arising from the slowest evolving regions of the bacterial genome. The use of long-read technologies in the future may allow for more informative sites from repetitive regions to be included in the analysis, which may improve the applicability and resolution of WGS in epidemiology.

## 11. WGS Provides New Insights into the Global Distribution of *M. bovis* Lineages

In the past years, WGS has helped define MTBC lineages, particularly those adapted to humans (*M. tuberculosis* L1 through L4 and L7, and *M. africanum* L5 and L6) [258]. *Mycobacterium tuberculosis* and *M. africanum* global lineage distribution has been associated with geography and human populations, and later shown to have distinct profiles of virulence and drug resistance acquisition [258,259]. Similar attempts to classify *M. bovis* genetically have been made by using a limited set of markers, leading to the classification of clonal complexes (CCs). Accordingly, four *M. bovis* CCs have been described (African 1 and 2, European 1 and 2), and these are determined based on specific deletions ranging from 806 to 14,094 bp, few SNPs and spoligotypes [260,261,262,263]. Similarly to *M. tuberculosis* lineages, CCs appear to be geographically segregated, with African 1 and 2 restricted to Africa, European 2 usually found in the Iberian Peninsula, and European 1 distributed globally [118,260,261,262,263]. However, *M. bovis* WGS studies indicate that not all isolates can be classified into these complexes, indicating that CCs do not represent the whole genetic diversity of *M. bovis* [21,48,161]. More recently, a global collection of 1,969 *M. bovis* genomes from different countries has been analyzed using whole-genome based phylogenetics [45]. This study proposed the existence of at least four distinct global lineages of *M. bovis* (Lb1 to Lb4), geographically segregated and not fully represented by CCs. There were still few *M. bovis* genomes without CC markers that could not be classified in any of these lineages (unknown clusters 1, 2 and 3) [45]. Another study also described *M. bovis* isolates without CC classification in France and suggested that these might be country-specific lineages [228]. As these French *M. bovis* genomes have not been compared to global genome collections, their lineage classification remains to be unraveled. As more *M. bovis* genomes are sequenced in the future, particularly from Africa and Asia, a more complete picture of *M. bovis* lineages global distribution will be determined. The continuous investigation of *M. bovis* genomes at the global level will provide opportunities to understand differences in virulence and transmission profiles underlying the current disease distribution.

## 12. Other Pathogens Causing bTB

*Mycobacterium caprae* is a causative agent of TB in animals of the *Bovidae* family [264,265,266]. This pathogen has been mostly detected in the European continent, with few reports of *M. caprae* in animals outside of Europe and cases of zoonotic TB in European patients detected in other countries. Accordingly, one strain of *M. caprae* was isolated from cattle in Algeria but has been linked to a possible introduction from mainland Europe [267]. In Morocco, three isolates of animal MTBC with intact RD4 and *M. caprae*-associated spoligotype were obtained from cattle [268], and in Japan, one captive Borneo elephant was found infected with *M. caprae* [269]. With a similar generalist tropism for hosts compared to *M. bovis*, *M. caprae* has been isolated from humans, goats, sheep, cattle, pigs, red deer (*Cervus elaphus*), wild boars (*Sus scrofa*), foxes (*Vulpes vulpes*), European bisons (*Bison bonasus*), Borneo elephant, and captive dromedary camel (*Camelus dromedarius*) [264,265,266]. In Spain, the number of cattle farms from which *M. caprae* was isolated accounted for 0.85–6.67% of the total number of herds with bTB, a number that is increasing over years [264]. WGS has been successfully used for contact tracing of *M. caprae* in cattle herds from Germany, showing evidence of within and between farm transmission [44].

More recently, the possibility of *M. orygis* as a primary pathogen species causing bTB in South Asia has been raised due to the observation of TB caused by this species in people from the region [192,270]. However, very little is known about the true host range of *M. orygis*, as it has been isolated from cattle, oryxes, gazelles, deer, antelope, waterbucks, and non-human primates [271]. A single outbreak of *M. orygis* in a dairy farm of mixed-breed animals of *Bos taurus* (Friesian breed) and *Bos taurus indicus* (Sahiwal breed), with 18 affected animals was reported [272]. As similar outbreaks in alternative species are also described for *M. tuberculosis* (e.g., elephants) and *M. bovis* (e.g., dogs) [273,274,275,276], further studies should be conducted on the actual host range of *M. orygis* and if cattle is a reservoir for this bacterial species.

## 13. Conclusions and Perspectives

In this review, we outlined current standards and/or challenges that remain to be unraveled on genotyping and WGS of *M. bovis* as tools for epidemiologic investigations. One important step towards implementation of WGS in programs of bTB control and eradication is certainly the standardization of data analysis and reporting of *M. bovis* WGS outcome. Research gaps associated with these subjects have been identified and described throughout this review (Table 1). Although continuous efforts must be made to address these challenges, WGS ultimate implementation in bTB programs must also integrate systems administration, management of resulting databases, and maintenance of the pipeline. Another important aspect of standardizing data generation and analysis is to define sets of *M. bovis* isolates and genomes that can be used for validation of different approaches as well as between laboratories.

The field of bTB has unquestionably experienced many technique advancements for transmission investigation and surveillance, from genotyping to genome sequencing. Yet, the disease remains a significant challenge in numerous parts of the world. Many low-to-middle income countries have still to establish basic disease control and eradication programs, and they have not benefited from *M. bovis* genotyping in the past. Only few, developed countries, with well-established bTB control programs, have implemented *M. bovis* genotyping as an epidemiologic tool. In addition, many genotyping studies worldwide have been performed in a retrospective, research-oriented manner, frequently not providing real-time investigation to solve outbreaks. Nevertheless, these studies have been incredibly valuable to understand transmission dynamics at the local and country levels, providing important information for public policy implementations. Not surprisingly, the same developed countries with a tradition in applying genotyping techniques into their bTB programs have overcome barriers to apply *M. bovis* WGS in their transmission investigations or on a research-basis, such as the USA, Ireland, New Zealand, and France [21,43,46,50,51,202,210,212,228]. Data generated from these countries and beyond show that WGS provides superior resolution power when compared to traditional genotyping techniques. In addition, WGS provided the means to evaluate the global structure of *M. bovis* population, bringing valuable insights into the current disease distribution [45].

It is evident that the research community has proven the usefulness of genotyping techniques for *M. bovis* transmission detection and surveillance and is now accumulating evidence on the applicability of WGS for the same purposes. However, compared to genotyping, WGS will likely see a much slower pace of employment in bTB programs and research. The requirement for an articulate bTB control and eradication program, specialized personnel, laboratory and computing infra-structure, good internet connectivity, streamlined operational procedures and protocols for data generation, availability of reagents, bioinformatic pipelines, and integrated and effective veterinary services are obstacles for widespread *M. bovis* WGS implementation in many countries [26,34,277]. In addition, despite continuous drops in prices, WGS can still reach a high-cost per sample, especially if just a few isolates need to be sequenced [26]. Thus, successful implementation of *M. bovis* WGS depends on multiple factors and will be contingent on the veterinary service strength, country-specific willingness to eradicate and control bTB, and investments. Most importantly, current stakeholders have to understand the value of such tools in controlling the disease, and this requires continuous research in different scenarios showing its applicability to resolve outbreaks.

## Figures and Tables

**Table 1:** Proposed research gaps and areas that need further development and exploration.

Pipeline Step	Areas in Need of Further Exploration
Bacterial isolation and sequencing	Methodologies to assess the possibility of cross-contamination with MTBC isolates
Quality assessment of entry data (FASTQ)	Comparison of protocols with different parameters or stringency levels of read trimming and filtering, reference mapping, removal of PCR duplicates, minimum acceptable median read length, contaminants handling, etc. *
Read processing	Choice of reference genome Parameters of read mapping (e.g., realignment around indels) Parameters of variant calling How to handle low quality variant calls How to detect and handle variants within repetitive areas Methodologies for detection of mixed-sample (number of reads supporting an allele and number of acceptable heterozygous sites based on established parameters of variant calling)
Transmission cluster detection	Comparison and/or development of different approaches: SNP-count, cgMLST, pgMLST, phylogenetic inferences
Data reporting	Standardization of WGS data reporting to end-users
Validation and inter-laboratory quality control	Validation datasets (of bacterial isolates and genomes) Protocols for inter-laboratory standardization (from bacterial isolation to sequencing)

* Technical validations should encompass the impact of choosing different parameters or stringency levels on the analysis output tailored for each need (contact investigation, surveillance, drug resistance detection), and also the relevance of these steps in the final outcome (are all these steps and parameters necessary to achieve the correct outcome?).

## Supplementary Materials

The following are available online at https://www.mdpi.com/2076-2607/8/5/667/s1, Table S1: List of research articles comparing traditional DNA typing methods using *Mycobacterium bovis* isolates (1998 to mid-2019), Table S2: Characteristics of genotyping techniques and whole-genome sequencing (WGS) used in *Mycobacterium bovis* studies, Table S3: Characteristics of whole genome sequencing studies of *Mycobacterium bovis.* Figure S1: Schematic representation of the genomic regions involved in genotyping techniques used in *Mycobacterium bovis* studies.
