# Supplementary material for: Mycobacterium bovis: From Genotyping to Genome Sequencing
Source: Microorganisms. 2020 May 3;8(5):667. doi: 10.3390/microorganisms8050667 (PMC7285088; doi:10.3390/microorganisms8050667)
Supplement: Supplementary file 1 [file microorganisms-08-00667-s001.zip › Table_S1.docx]

**Methods**

In July 2019, we searched PubMed for the keywords “Mycobacterium bovis” and “spoligotyping”. A total of 235 articles were found. Of these, we downloaded all articles pertaining epidemiological studies using *M. bovis* isolates, totalizing 206 articles (e.g. excluding articles that were using *M. bovis* as reference strain for comparison against *M. tuberculosis*, or that were related to single case reports in humans or animals). From these, we identified 38 articles that compared different DNA typing methods, which are compiled in the table below (Table S1). We did not search for “Mycobacterium bovis” and “VNTR” as to avoid retrieving articles that only evaluated different panels of VNTR loci, as our focus was to compare distinct DNA typing techniques. To provide more recent data, we chose the term “spoligotyping” to fix it in our analysis (i.e. all articles had *M. bovis* isolates evaluated by spoligotyping).

**Table S1.** List of research articles comparing traditional DNA typing methods using *Mycobacterium bovis* isolates (1998 – mid-2019).

|  | **Article**  **(by year)** | **Country** | **number of isolates** | **Number of types identified** | | | | | | **Information/quotes extracted from the article** |
| --- | --- | --- | --- | --- | --- | --- | --- | --- | --- | --- |
|  |  |  |  | **REA** | **IS6110-RFLP** | **PGRS-RFLP** | **DR-RFLP** | **Spoligo** | **VNTR** |  |
| **1** | Cousins et al., 1998 | Australia, Canada, Ireland | 273 | - | 23 | 77 | 35 | 35 | - | “RFLP-IS6110 was sufficiently sensitive for the typing of isolates with more than three copies of IS6110, but RFLP-PGRS probe was the most sensitive typing technique for strains with only a single copy of IS6110”. |
| **2** | Roring et al., 1998 | Ireland | 47 | - | 15 | 15 | 12 | 15 | - | Combined = 24 RFLP types, which was more discriminatory than Spoligotyping. But Spoligotyping was faster and easier to perform. |
| **3** | Aranaz et al., 1998 | Spain | 128 | - | 25 | 28 | 17 | 19 | - | “The most sensitive technique for identifying polymorphism in *M. bovis* was PGRS-RFLP, closely followed by IS6110-RFLP”. |
| **4** | Collins, 1999 | New Zealand | 331 | 26 | - | 11 | - | 7 | - |  |
| **5** | Zumárraga et al., 1999 | Argentina, Uruguay, Paraguay, Mexico, Costa Rica | 154 | - | - | 42 | 42 | 31 | - | “Although the differentiation of *M. bovis* by spoligotyping was less discriminatory than differentiation by RFLP analysis with the DR and PGRS probes, spoligotyping is easier to perform and its results are easier to interpret”. |
| **6** | Costello et al., 1999 | Ireland | 452 | - | 36 | 48 | 23 | 20 | - | Combined = 85 RFLP types. “The PGRS probe gave better differentiation of strains than the IS6110 or DR probes. The majority 6of isolates from all s7pecies carried a single IS86110 copy”. |
| **7** | Skuce et al., 2002 | UK | 100 | - | - | - | - | 29 | 22 (ETR-A-E)  33 (7-loci: QUB-5,11a,11b,15,18,23,26) | Combined = 51 VNTR types. Spoligotyping HGDI of 0.79; VNTR HGDI of 0.96. “The novel VNTR targets (QUB) identified in this study should additionally increase the power of this approach”. |
| **8** | Cowan et al., 2002 | USA (Michigan) | 180^a^ | - | 58 | - | - | 59 | 80 (12-loci, MIRU) | Combined (all) = 112 types. “The results confirm the potential utility of MIRU-VNTR typing and show that typing with multiple methods is required to attain maximum specificity”. |
| **9** | Roring et al., 2002 | Ireland | 47 | - | - | - | - | 11 | 10 (8 loci: 0960c, 2531c, QUB 3232, 1451, 1895, 4156c, 3336, 1281c)  6 (ETR-A-E)  14 (ETR-A, B, QUB 3232, 1895, 3336) | Combined = 14 VNTR types. Spoligotyping HGDI of 0.66; VNTR HGDI of 0.87. “The novel VNTR markers described increased the discrimination possible in strain typing of *M. bovis*, with the added benefit of an intuitive digital nomenclature”. |
| **10** | Roring et al., 2004 | Ireland | 47 | - | - | - | - | 11 | 6 (ETR-A-E)  11 (12 MIRU loci)  17 (6 QUB loci)  10 (5 QUB loci)  3 (4 QUB loci) | Combination of 18 best discriminating loci = 36 VNTR types. “An optimum set of 10 VNTRs, with a discrimination index of 0.95 was found. This VNTR panel (VNTRs 11a, 11b, 26, 1895, 3232, 3336, ETR-A, ETR-B, MIRUs 24 and 26), resolved the panel of *M. bovis* isolates into 30 allele profiles (h = 0.96).” |
| **11** | Zanini et al., 2005 | Brazil | 163 | - | 9 | 13^b^ | - | 12^b^ | - | A comparison between the genotype data obtained fails to show a correlation between the main clusters found by the three techniques. |
| **12** | Hilty et al., 2005 | Chad | 67 | - | - | - | - | 16 | 22 (ETR-A-C)  18 (12 MIRU) | 16 VNTR loci = 12 MIRU, 3 ETRs and VNTR 3232. Combined (all) = 33 VNTR types. “VNTR typing was highly discriminative with an overall allelic diversity (h_oa_) of 0.922.” “VNTR typing of the five highly discriminative loci (ETR-A-C, MIRUs 26, 27) (h = 0.917) proved to be most appropriate for first line typing of *M. bovis* strains of Chad and superior than spoligotyping (h_sp_ = 0.789).” |
| **13** | Skuce et al., 2005 | Ireland | 461 | - | - | - | - | 14 | 40 (7 loci: QUB 3232, 11a, 1895, 26a, 11b, ETR-A-B) | Combined (all) = 43 types (HGDI of 0.899). Spoligotyping HGDI of 0.533; VNTR HGDI of 0.897. |
| **14** | Razanamparany et al., 2006 | Madagascar | 180 | - | 9 | - | 20 | 12 | - | Combined (all) – 34 types. “DR was the most discriminatory of all three markers”. |
| **15** | Allix et al., 2006 | Belgium | 68 | - | 16 | - | - | 17 | 32 (29 MIRU-VNTR loci) | Combined (all) = 35 types. Spoligotyping HGDI of 0.85; MIRU-VNTR HGDI of 0.91; IS6110-RFLP HGDI of 0.73; all combined HGDI of 0.94. “…Maximal resolution was already achieved with a subset of 9 loci. ” |
| **16** | Michel et al., 2008 | South Africa | 91 | - | 16^c^ | 18^c^ | - | 12^c^ | 13^c^ | Combined (all) = 29 types. “PGRS RFLP was the single most discriminatory method and combinations of typing methods, which included IS6110 and/or PGRS had the highest discriminatory power, able to reveal 29 distinct genotypes among 35 farms with no epidemiological link.” |
| **17** | Romero et al., 2008 | Spain | 163 | - | - | - | - | 9 | 8 (8 loci: QUB3232, 26, 11a, 11b, 4, 40, ETR-A-B, MIRU 4, 40)^d^ | Spoligotyping HGDI of 0.38; VNTR HGDI of 0.46. |
| **18** | Martinez et al., 2008 | Mexico, USA | 41 | - | - | - | - | 11 | 21 (27 VNTR loci) | “MLVA genotyping of *M. bovis* shows great potential as a molecular typing tool for characterizing the epidemiology of *M. bovis* animal infections in North America. However, the greatest resolution was achieved by using a combination of both MLVA and spoligotyping”. |
| **19** | Boniotti et al., 2009 | Italy | 1,503 | - | - | - | - | 81 | 11 (ETR-A-E)  89 (24 MIRU-VNTR loci in SB0120 isolates)^e^ | Combined (all) = 228. “Despite the high degree of resolution obtained, the spoligotyping/ETR methods were not discriminative enough in the case of genotypes characterized by the combination of SB0120, the predominant spoligotype in Italy, with the most common ETR profiles.” A minimum panel of 13 loci was proposed. |
| **20** | Duarte et al., 2010 | Portugal | 181^f^ | - | - | - | - | 12 | 87 (8 loci: VNTR 3232, ETR-A-C, QUB 11b,b, MIRU 4, 26) | VNTR HGDI of 0.99. “MIRU-VNTR typing was superior to spoligotyping for identifying multi-genotype infected herds and the combination of the two genotyping methods by a hierarchical approach confirmed the genetic relatedness of *M. bovis* isolates between cattle and wildlife”. |
| **21** | McLernon et al., 2010 | Ireland | 386 | - | * | * | * | 15 | 41 (6 loci: QUB 11a,b, ETR A, 4052, MIRU 26, 1895). | * RFLP (IS6110, PGRS, DR) was combined into one class = 65 types. “RFLP analysis was the method that gave the greatest differentiation of strains, with a HGDI of 0.927; the HGDI recorded for MIRU-VNTR typing was marginally lower at 0.918, and spoligotyping was the least discriminatory method, with an HGDI of 0.7.” |
| **22** | Lari et al., 2011 | Italy | 47^g^ | - | - | - | - | 15 | 29 (15 VNTR loci) | Combined (all) = 33 types. |
| **23** | Price-Carter et al., 2011 | New Zealand | 204 | 68 | - | - | - | - | 33 (22 VNTR loci + 2 DR-based PCR assays) | Additional testing can be found in the article: 45 VNTR loci were investigated in 78 isolates representing the 26 most common REA types in New Zealand. The same isolates were subjected to two newly designed DR-based PCR to resolve REA 219. Finally, 204 isolates representing 68 REA types were analyzed by 22 VNTR loci and two DR-based PCR assays. “Major differences were found in allelic variation of some VNTRs between isolates from New Zealand and other countries, emphasizing the importance of adapting *M. bovis* typing systems to suit individual countries.” |
| **24** | Sun et al., 2012 | China | 135 | - | - | - | - | 4 | 7 (24 MIRU-VNTR loci) | “A new combination of nine MIRU-VNTR loci was most discriminative for *M. bovis* clones from Xinjiang.” – with a HGDI of 0.62. |
| **25** | Rodriguez-Campos et al., 2011 | Spain | 47 | - | - | - | - | 3 | 11 (9 loci: ETR-A, B, D, E, MIRU 26, QUB 11a, b, 26, 3232) | Spoligotyping + VNTR HGDI of 0.889. There was a high genotypic diversity which precluded the identification of the source of infection to an alpaca herd. |
| **26** | Figueiredo et al., 2012 | Brazil | 12 | - | - | - | - | 4 | 10 (15 loci: 12 MIRU, ETR-A-C) | A single outbreak was analyzed, revealing a high genetic diversity suggestive of multiple pathogen introductions. “MIRU loci 4 and 26, as well as ETR (A, B, C), were highly discriminative” |
| **27** | Parreiras et al., 2012 | Brazil | 61 | - | - | - | - | 17 | 16 (12 MIRU-VNTR loci) | Combined (all) = 29 types. “…only two of the 12-MIRU-VNTR loci presented scores with either a moderate (0.4, MIRU16) or high (0.6, MIRU26) discriminatory index (h). Both typing methods produced similar discriminatory indexes (spoligotyping h = 0.85; MIRU-VNTR h = 0.86) and the combination of the two methods increased the h value to 0.94, resulting in 29 distinct patterns.” |
| **28** | Furphy et al., 2012 | Ireland | 93 | - | - | - | - | 9 | 22 (6 loci: QUB11a, b, 26, ETR-A, MIRU 26, 1895) |  |
| **29** | Rocha et al., 2013 | Brazil | 116 | - | - | - | - | 5 | Not reported (10 MIRU-VNTR loci + ETR-A-C) | Spoligotyping HDGI of 0.74; MIRU-VNTR HGDI of 0.83; and ETR HGDI of 0.82. “The associations of the methods’ improved discriminatory power were: spoligotyping + MIRU = 0.93; spoligotyping + ETR = 0.93; and MIRU+ETR=0.95. The greatest discriminatory power was obtained when the three techniques were associated (HGI = 0.98051).” |
| **30** | Lamine-Khemiri et al., 2014 | Tunisia | 35 | - | - | - | - | 9 | 19 (6 loci: ETR-A, B, D, QUB11a, b, 3232) | Spoligotyping HGDI of 0.753; VNTR HGDI of 0.969. Two isolates were identified as *M. caprae based* on spoligotyping. |
| **31** | Ramos et al., 2014 | Brazil | 85 | - | - | - | - | 4 | 5 (5 loci: ETR-A,B, QUB 1895,3336, MIRU 26) | Spoligotyping HGDI of 0.14; VNTR HGDI of 0.66. |
| **32** | Biffa et al., 2014 | Ethiopia | 58 | - | - | - | - | 17 | 19 (28 VNTR loci) | Spoligotyping HGDI of 0.82; VNTR HGDI of 0.92. Adoption of the nine VNTR loci with >3 alleles provided good differentiation between the isolates. |
| **33** | Hauer et al., 2015 | France | 4,654 | - | - | - | - | 176 | 498 (8 MIRU-VNTR loci) | MIRU-VNTR HDGI of 0.47-0.981 depending on the Spoligotype. “three spoligotypes are predominant and account for more than half of the total strain population: SB0120 (26%), SB0134 (11%) and SB0121 (6%).” |
| **34** | Carvalho et al., 2016 | Brazil | 37 | - | - | - | - | 10 | 28 (24 MIRU-VNTR loci) | Spoligotyping HGDI of 0.81; VNTR HGDI of 0.98. “…ETR-A and QUB 11b loci, showed high discriminatory ability (h = 0.50), while MIRU 16, MIRU 27, ETR-B, ETR-C, Mtub21 and QUB 26 loci showed moderate ability (h = 0.33 or h = 0.49).” |
| **35** | Hauer et al., 2016 | France | 2,332 | - | - | - | - | 153 | 471 (8 MIRU-VNTR loci) | Spoligotyping HGDI of 0.882; VNTR HGDI of 0.98. “…69% of the strains are grouped in only ten spoligotypes of which three are dominant: SB0120, SB0134 and SB0121 (30, 12 and 6% respectively)”. “Differences in global DI per spoligotype, but also by locus within each spoligotype, were observed.” “…the loci employed for MLVA in a country should be those which are the most discriminative for the clonal complexes which characterize their *M. bovis* population”. |
| **36** | Ghielmetti et al., 2017 | Switzerland | 24^h^ | - | - | - | - | 2 | 4 (49 VNTR loci) | Investigation of a single outbreak corresponding to the re-emergence of bTB in Switzerland. |
| **37** | Egbe et al., 2017 | Cameroon | 225 | - | - | - | - | 37 | 97 (24 MIRU-VNTR loci) | Spoligotyping HGDI of 0.801; Spoligotyping + MIRU-VNTR HGDI of 0.982. “Two of the 24 loci, ETR C, QUB-26, were highly discriminatory (h > 0.55) while four loci, which include ETR A, ETR B, QUB-11b and MIRU-26 were considered moderately discriminatory (0.33 < h < 0.55).” |
| **38** | Armas et al., 2017 | Italy (Sicily) | 49 | - | - | - | - | 5 | 17 (12 MIRU-VNTR loci) | Spoligotyping HGDI of 0.433; VNTR HGDI of 0.873. Ten types were identified with rep-PCR[39] and 4 with *embB* polymorphisms. |

^a^180 low-copy IS6110 isolates of *M. tuberculosis* and *M. bovis*. ^b^only 16 (PGRS-RFLP) or 36 (Spoligotyping) out of the 163 *M. bovis* isolates were tested. ^c^only 49 (IS6110-RFLP), 25 (PGRS-RFLP), 50 (Spoligotyping) or 43 (VNTR) out of the 91 *M. bovis* isolates were tested. ^d^92 out of the 163 *M. bovis* isolates were tested. ^e^100 SB0120 *M. bovis* isolates were tested. ^f^*M. bovis* and *M. caprae* isolates. ^g^Nine BCG, 37 *M. bovis* and 1 M. caprae isolates obtained from humans in Tuscany, Italy. ^h^Seventeen *M. bovis* and seven *M. caprae*. HGDI or h or DI: Hunter and Gaston discriminatory index [40]. MLVA: Multi-locus variable-number tandem repeat analysis. VNTR: Variable number of tandem repeats. RFLP: Restriction fragment length polymorphism. -: not done.

**References**

1. Cousins, D.; Williams, S.; Liébana, E.; Aranaz, A.; Bunschoten, A.; Van Embden, J.; Ellis, T. Evaluation of four DNA typing techniques in epidemiological investigations of bovine tuberculosis. *J. Clin. Microbiol.* **1998**, *36*, 168–178.

2. Roring, S.; Brittain, D.; Bunschoten, A.E.; Hughes, M.S.; Skuce, R.A.; Van Embden, J.D.A.; Neill, S.D. Spacer oligotyping of Mycobacterium bovis isolates compared to typing by restriction fragment length polymorphism using PGRS, DR and IS6110 probes. *Vet. Microbiol.* **1998**, *61*, 111–120.

3. Aranaz, A.; Liébana, E.; Mateos, A.; Domínguez, L.; Cousins, D. Restriction fragment length polymorphism and spacer oligonucleotide typing: A comparative analysis of fingerprinting strategies for Mycobacterium bovis. *Vet. Microbiol.* **1998**, *61*, 311–324.

4. Collins, D.M. DNA typing of Mycobacterium bovis strains from the castlepoint area of the Wairarapa. *N. Z. Vet. J.* **1999**, *47*, 207–209.

5. Zumárraga, M.J.; Martin, C.; Samper, S.; Alito, A.; Latini, O.; Bigi, F.; Roxo, E.; Cicuta, M.E.; Errico, F.; Ramos, M.C.; et al. Usefulness of spoligotyping in molecular epidemiology of Mycobacterium bovis-related infections in South America. *J. Clin. Microbiol.* **1999**, *37*, 296–303.

6. Costello, E.; O’Grady, D.; Flynn, O.; O’Brien, R.; Rogers, M.; Quigley, F.; Egan, J.; Griffin, J. Study of restriction fragment length polymorphism analysis and spoligotyping for epidemiological investigation of Mycobacterium bovis infection. *J. Clin. Microbiol.* **1999**, *37*, 3217–3222.

7. Skuce, R.A.; McCorry, T.P.; McCarroll, J.F.; Roring, S.M.M.; Scott, A.N.; Brittain, D.; Hughes, S.L.; Hewinson, R.G.; Neill, S.D. Discrimination of Mycobacterium tuberculosis complex bacteria using novel VNTR-PCR targets. *Microbiology* **2002**, *148*, 519–528.

8. Cowan, L.S.; Mosher, L.; Diem, L.; Massey, J.P.; Crawford, J.T. Variable-number tandem repeat typing of Mycobacterium tuberculosis isolates with low copy numbers of IS6110 by using mycobacterial interspersed repetitive units. *J. Clin. Microbiol.* **2002**, *40*, 1592–1602.

9. Roring, S.; Scott, A.; Brittain, D.; Walker, I.; Hewinson, G.; Neill, S.; Skuce, R. Development of Variable-Number Tandem Repeat Typing of Mycobacterium bovis: Comparison of Results with Those Obtained by Using Existing Exact Tandem Repeats and Spoligotyping. *J. Clin. Microbiol.* **2002**, *40*, 2126–2133.

10. Roring, S.; Scott, A.N.; Hewinson, R.G.; Neill, S.D.; Skuce, R.A. Evaluation of variable number tandem repeat (VNTR) loci in molecular typing of Mycobacterium bovis isolates from Ireland. *Vet. Microbiol.* **2004**, *101*, 65–73.

11. Zanini, M.S.; Moreira, E.C.; Salas, C.E.; Lopes, M.T.P.; Barouni, A.S.; Roxo, E.; Telles, M.A.; Zumarraga, M.J. Molecular typing of Mycobacterium bovis isolates from south-east Brazil by spoligotyping and RFLP. *J. Vet. Med. Ser. B Infect. Dis. Vet. Public Heal.* **2005**, *52*, 129–133.

12. Hilty, M.; Diguimbaye, C.; Schelling, E.; Baggi, F.; Tanner, M.; Zinsstag, J. Evaluation of the discriminatory power of variable number tandem repeat (VNTR) typing of Mycobacterium bovis strains. *Vet. Microbiol.* **2005**, *109*, 217–222.

13. Skuce, R.A.; McDowell, S.W.; Mallon, T.R.; Luke, B.; Breadon, E.L.; Lagan, P.L.; McCormick, C.M.; McBride, S.H.; Pollock, J.M. Discrimination of isolates of Myobacterium bovis in Northern Ireland on the basis of variable numbers of tandem repeates (VNTRs). *Vet. Rec.* **2005**, *157*, 501–504.

14. Razanamparany, V.R.; Quirin, R.; Rapaoliarijaona, A.; Rakotoaritahina, H.; Vololonirina, E.J.; Rasolonavalona, T.; Ferdinand, S.; Sola, C.; Rastogi, N.; Ramarokoto, H.; et al. Usefulness of restriction fragment length polymorphism and spoligotyping for epidemiological studies of Mycobacterium bovis in Madagascar: Description of new genotypes. *Vet. Microbiol.* **2006**, *114*, 115–122.

15. Allix, C.; Walravens, K.; Saegerman, C.; Godfroid, J.; Supply, P.; Fauville-Dufaux, M. Evaluation of the epidemiological relevance of variable-number tandem-repeat genotyping of Mycobacterium bovis and comparison of the method with IS6110 restriction fragment length polymorphism analysis and spoligotyping. *J. Clin. Microbiol.* **2006**, *44*, 1951–1962.

16. Michel, A.L.; Hlokwe, T.M.; Coetzee, M.L.; Maré, L.; Connoway, L.; Rutten, V.P.M.G.; Kremer, K. High Mycobacterium bovis genetic diversity in a low prevalence setting. *Vet. Microbiol.* **2008**, *126*, 151–159.

17. Romero, B.; Aranaz, A.; Sandoval, Á.; Álvarez, J.; de Juan, L.; Bezos, J.; Sánchez, C.; Galka, M.; Fernández, P.; Mateos, A.; et al. Persistence and molecular evolution of Mycobacterium bovis population from cattle and wildlife in Doñana National Park revealed by genotype variation. *Vet. Microbiol.* **2008**, *132*, 87–95.

18. Martinez, L.R.; Harris, B.; Black, W.C.; Meyer, R.M.; Brennan, P.J.; Vissa, V.D.; Jones, R.L. Genotyping North American Animal Mycobacterium Bovis Isolates Using Multilocus Variable Number Tandem Repeat Analysis. *J. Vet. Diagnostic Investig.* **2008**, *20*, 707–715.

19. Boniotti, M.B.; Goria, M.; Loda, D.; Garrone, A.; Benedetto, A.; Mondo, A.; Tisato, E.; Zanoni, M.; Zoppi, S.; Dondo, A.; et al. Molecular typing of Mycobacterium bovis strains isolated in Italy from 2000 to 2006 and evaluation of variable-number tandem repeats for geographically optimized genotyping. *J. Clin. Microbiol.* **2009**, *47*, 636–644.

20. Duarte, E.L.; Domingos, M.; Amado, A.; Cunha, M. V.; Botelho, A. MIRU-VNTR typing adds discriminatory value to groups of Mycobacterium bovis and Mycobacterium caprae strains defined by spoligotyping. *Vet. Microbiol.* **2010**, *143*, 299–306.

21. McLernon, J.; Costello, E.; Flynn, O.; Madigan, G.; Ryan, F. Evaluation of mycobacterial interspersed repetitive-unit-variable-number tandem-repeat analysis and spoligotyping for genotyping of Mycobacterium bovis isolates and a comparison with restriction fragment length polymorphism typing. *J. Clin. Microbiol.* **2010**, *48*, 4541–4545.

22. Lari, N.; Bimbi, N.; Rindi, L.; Tortoli, E.; Garzelli, C. Genetic diversity of human isolates of Mycobacterium bovis assessed by spoligotyping and Variable Number Tandem Repeat genotyping. *Infect. Genet. Evol.* **2011**, *11*, 175–180.

23. Price-Carter, M.; Rooker, S.; Collins, D.M. Comparison of 45 variable number tandem repeat (VNTR) and two direct repeat (DR) assays to restriction endonuclease analysis for typing isolates of Mycobacterium bovis. *Vet. Microbiol.* **2011**, *150*, 107–114.

24. Sun, Z.; Cao, R.; Tian, M.; Zhang, X.; Zhang, X.; Li, Y.; Xu, Y.; Fan, W.; Huang, B.; Li, C. Evaluation of Spoligotyping and MIRU-VNTR for Mycobacterium bovis in Xinjiang, China. *Res. Vet. Sci.* **2012**, *92*, 236–239.

25. Rodriguez-Campos, S.; Aranaz, A.; De Juan, L.; Sáez-Llorente, J.L.; Romero, B.; Bezos, J.; Jiménez, A.; Mateos, A.; Domínguez, L. Limitations of spoligotyping and variable-number tandem-repeat typing for molecular tracing of Mycobacterium bovis in a high-diversity setting. *J. Clin. Microbiol.* **2011**, *49*, 3361–3364.

26. Figueiredo, E.E. de S.; Ramos, D.F.; Medeiros, L.; Silvestre, F.G.; Lilenbaum, W.; Silva, J.T.; Paschoalin, V.M.F.; Dellagostin, O.A. Multiple strains of Mycobacterium bovis revealed by molecular typing in a herd of cattle. *Vet. J.* **2012**, *193*, 296–298.

27. Parreiras, P.M.; Andrade, G.I.; do Nascimento, T. de F.; Oelemann, M.C.; Gomes, H.M.; de Alencar, A.P.; de Assis, R.A.; Coelho Mota, P.M.P.; Pereira, M.A. da S.; Lobato, F.C.F.; et al. Spoligotyping and variable number tandem repeat analysis of Mycobacterium bovis isolates from cattle in Brazil. *Mem. Inst. Oswaldo Cruz* **2012**, *107*, 64–73.

28. Furphy, C.; Costello, E.; Murphy, D.; Corner, L.A.L.; Gormley, E. DNA typing of mycobacterium bovis isolates from badgers (Meles meles) culled from areas in ireland with different levels of tuberculosis prevalence. *Vet. Med. Int.* **2012**, *2012*.

29. Rocha, V.C.F.; De Figueiredo, S.C.; Rosales, C.A.R.; De Hildebrand E Grisi Filho, J.H.; Keid, L.B.; Soares, R.M.; Ferreira Neto, J.S. Molecular discrimination of mycobacterium bovis in São Paulo, Brazil. *Vector-Borne Zoonotic Dis.* **2013**, *13*, 17–21.

30. Lamine-Khemiri, H.; Martínez, R.; García-Jiménez, W.L.; Benítez-Medina, J.M.; Cortés, M.; Hurtado, I.; Abassi, M.S.; Khazri, I.; Benzarti, M.; Hermoso-de-Mendoza, J. Genotypic characterization by spoligotyping and VNTR typing of Mycobacterium bovis and Mycobacterium caprae isolates from cattle of Tunisia. *Trop. Anim. Health Prod.* **2014**, *46*, 305–311.

31. Ramos, D.F.; Silva, A.B.S.; Fagundes, M.Q.; von Groll, A.; da Silva, P.E.A.; Dellagostin, O.A. Molecular typing of Mycobacterium bovis isolated in the south of Brazil. *Brazilian J. Microbiol.* **2014**, *45*, 657–660.

32. Biffa, D.; Johansen, T.B.; Godfroid, J.; Muwonge, A.; Skjerve, E.; Djønne, B. Multi-locus variable-number tandem repeat analysis (MLVA) reveals heterogeneity of Mycobacterium bovis strains and multiple genotype infections of cattle in Ethiopia. *Infect. Genet. Evol.* **2014**, *23*, 13–19.

33. Hauer, A.; De Cruz, K.; Cochard, T.; Godreuil, S.; Karoui, C.; Henault, S.; Bulach, T.; Bañuls, A.L.; Biet, F.; Boschiroli, M.L. Genetic evolution of mycobacterium bovis causing tuberculosis in livestock and wildlife in France since 1978. *PLoS One* **2015**, *10*, 1–17.

34. Carvalho, R.C.T.; Vasconcellos, S.E.G.; Issa, M.D.A.; Filho, P.M.S.; Mota, P.M.P.C.; De Araújo, F.R.; Carvalho, A.C.D.S.; Magdiniergomes, H.; Suffys, P.N.; Figueiredo, E.E.D.S.; et al. Molecular typing of Mycobacterium bovis from cattle reared in midwest Brazil. *PLoS One* **2016**, *11*, 1–16.

35. Hauer, A.; Michelet, L.; De Cruz, K.; Cochard, T.; Branger, M.; Karoui, C.; Henault, S.; Biet, F.; Boschiroli, M.L. MIRU-VNTR allelic variability depends on Mycobacterium bovis clonal group identity. *Infect. Genet. Evol.* **2016**, *45*, 165–169.

36. Ghielmetti, G.; Scherrer, S.; Friedel, U.; Frei, D.; Suter, D.; Perler, L.; Wittenbrink, M.M. Epidemiological tracing of bovine tuberculosis in Switzerland, multilocus variable number of tandem repeat analysis of Mycobacterium bovis and Mycobacterium caprae. *PLoS One* **2017**, *12*, 1–15.

37. Egbe, N.F.; Muwonge, A.; Ndip, L.; Kelly, R.F.; Sander, M.; Tanya, V.; Ngwa, V.N.; Handel, I.G.; Novak, A.; Ngandalo, R.; et al. Molecular epidemiology of Mycobacterium bovis in Cameroon. *Sci. Rep.* **2017**, *7*, 1–17.

38. Armas, F.; Camperio, C.; Coltella, L.; Selvaggini, S.; Boniotti, M.B.; Pacciarini, M.L.; Lo Presti, V.D.M.; Marianelli, C. Comparison of semi-automated commercial Rep-PCR fingerprinting, spoligotyping, 12-locus MIRU-VNTR typing and single nucleotide polymorphism analysis of the embb gene as molecular typing tools for mycobacterium bovis. *J. Med. Microbiol.* **2017**, *66*, 1151–1157.

39. Cangelosi, G.A.; Freeman, R.J.; Lewis, K.N.; Livingston-Rosanoff, D.; Shah, K.S.; Milan, S.J.; Goldberg, S. V. Evaluation of a high-throughput repetitive-sequence-based PCR system for DNA fingerprinting of Mycobacterium tuberculosis and Mycobacterium avium complex strains. *J. Clin. Microbiol.* **2004**, *42*, 2685–2693.

40. Hunter, P.R.; Gaston, M.A. Numerical index of the discriminatory ability of typing systems: An application of Simpson’s index of diversity. *J. Clin. Microbiol.* **1988**, *26*, 2465–2466.
