# Supplementary material for: Mycobacterium bovis: From Genotyping to Genome Sequencing
Source: Microorganisms. 2020 May 3;8(5):667. doi: 10.3390/microorganisms8050667 (PMC7285088; doi:10.3390/microorganisms8050667)
Supplement: Supplementary file 1 [file microorganisms-08-00667-s001.zip › Table_S3.docx]

**Table S3.** Characteristics of whole genome sequencing studies of *Mycobacterium bovis.*

| **Article** | **Country** | **Number of isolates (host)^a^** | **Purpose** | **Reference genome** | **Read mapping** | **Variant calling** | **SNP interpretation** |
| --- | --- | --- | --- | --- | --- | --- | --- |
| Biek et al., 2012 [1] | Ireland | 31 (26 cattle and 5 badgers) | Transmission investigation | *M. bovis* AF2122/97 | Geneious | Geneious  Mauve | ML + SNP count |
| Trewby et al., 2016 [2] | Ireland | 144 (138 cattle and 6 badgers) | Transmission investigation | *M. bovis* AF2122/97 | BWA | SAMTools | ML + Bayesian inference + Pair-wise SNP distance |
| Acosta et al., 2015 [3] | Panama | 7 (cattle) | Source investigation | *M. bovis* AF2122/97 | Bowtie2 | VCFtools | UPGMA^c^ |
| Glaser et al., 2016 [4] | USA | 65 (34 cattle and 31 deer) | Transmission and source investigation + surveillance | *M. bovis* AF2122/97 | BWA | GATK (Unified genotyper) | ML + temporal analysis (BEAST) |
| Broeckl et al., 2017 [5] | Germany | 61 *M. caprae* (cattle) | Transmission investigation + surveillance | *M. caprae* | BWA | VarScan | ML + SNP count + median-joining networks |
| Sandoval-Azuara t al., 2017 [6] | Mexico | 172 (155 cattle and 17 humans) | Source investigation to humans + *M. bovis* populational structure | *M. bovis* AF2122/97 | BWA | GATK (Haplotype caller) | ML |
| Crispell et al., 2017 [7] | New Zealand | 296 (222 cattle and 74 wildlife) | Transmission investigation (role of wildlife) + populational structure + surveillance | *M. bovis* AF2122/97 | BWA | Not described | ML + Pair-wise genetic distance + |
| Patané et al., 2017 [8] | Global | 114 (from 14 host species) | Populational structure + genomic characteristics + evolution | NA | NA | NA | ML from core genes alignment |
| Dippenaar et al., 2017 [9] | South Africa | 106 (many host species – not specified) | To identify if *M. bovis* isolates cluster according to host or geography. | *M. tuberculosis* H37Rv | BWA  NovoAlign  SMALT | GATK^d^ | ML |
| Zimpel et al., 2017 [10] | Global | 31 *M. bovis* | Populational structure | NA | NA | kSNP3 | ML + Neighbor-Joining |
| Ghebremariam et al., 2018 [11] | Eritrea | 14 (cattle) | Populational structure + surveillance | *M. bovis* AF2122/97 | BWA | GATK (Haplotype caller) | ML |
| Kohl et al., 2018 [12] | Germany | 18 (captive wildlife) | Transmission. investigation + surveillance | *M. tuberculosis* H37Rv | BWA | SAMtools  Perls scripts | ML + MP + SNP count |
| Lasserre et al., 2018 [13] | Global | 186 (23 from Uruguay) | Populational structure | NA | NA | NA | ML from core genes alignment |
| Orloski et al., 2018 [14] | USA | 1,248 (cattle and cervids) | Transmission and source investigation + surveillance + populational structure + | *M. bovis* AF2122/97 | BWA | GTAK (Haplotype caller) | ML + SNP count |
| Otchere et al., 2019 [15] | Global | 772 | Populational structure | *M. bovis* AF2122/97 | BWA | SAMtools (BCFtools) | ML |
| Abdelaal et al., 2019 [16] | Egypt | 11 | Populational structure + surveillance | *M. bovis* AF2122/97 | CLC-Bio Genomic Workbench | CLC-Bio Genomic Workbench | Neighbor-Joining + temporal analysis (BEAST) |
| Salvador et al., 2019 [17] | USA | 134 (deer, elk and cattle) | Transmission investigation (role of wildlife) + surveillance | *M. bovis* AF2122/97 | BWA | GATK | Phylogenetic reconstruction and ancestral state/host (BEAST) + genetic distances |
| Zimpel t al., 2019 [18] | Global | 823 (cattle and wildlife) | Populational structure (lineages) | *M. bovis* AF2122/97 | BWA | VarScan2 | ML + MP + temporal analysis (BEAST) |
| Michelet et al., 2019 [19] | France | 24 (12 cattle, 12 wildlife) | Transmission investigation (role of wildlife) | *M. bovis* AF2122/97 | BioNumerics | BioNumerics | ML + SNP count |
| Crispell et al., 2019 [20] | Ireland | 352 (193 badger, 159 cattle) | Transmission investigation (role of wildlife) | *M. bovis* AF2122/97 | BWA | SAMtools (BCFtools) | Phylogenetic reconstruction, transmission rate estimation (BEAST) + SNP count |
| Anzai, 2019 [21] | Brazil | 23 (cattle) | Transmission investigation | *M. bovis* SP38 | BWA | Ridom SeqSphere+ | cgMLST |
| Hauer et al., 2019 [22] | France | 87 (cattle, sheep, pig, goat, wildlife) | Populational structure | *M. bovis* AF2122/97 | SMALT | SAMtools (BCFtools) | Neighbor-Joining + ML |

^a^*M. bovis* isolates, unless otherwise stated. ^b^Genomes were assembled using Geneious assembler and variants were called using Mauve. ^c^unweighted pair-grouping method analysis algorithm. ^d^Variants identified by the GATK in all three alignments were considered true. BEAST: Bayesian Evolutionary Analysis by Sampling Trees. Surveillance was defined when a temporal analysis was performed (either by using *M. bovis* isolates of different years, or by estimating date of pathogen introduction). NA: Not applicable. Patané et al., 2017 and Lasserre etal., 2018 used core gene alignments, derived from assembled genomes, to generate a phylogenetic tree using maximum likelihood. Zimpel et al., 2017 used assembled *M. bovis* genomes as input to kSNP3. Genome announcements were not included in this table. “Global” means that M. bovis genomes were gathered from multiple countries, normally accessing public databases (e.g. GenBank). ML: Maximum likelihood tree, MP: Maximum Parsimony tree.

**References**

1. Biek, R.; O’Hare, A.; Wright, D.; Mallon, T.; McCormick, C.; Orton, R.J.; McDowell, S.; Trewby, H.; Skuce, R.A.; Kao, R.R. Whole genome sequencing reveals local transmission patterns of *Mycobacterium bovis* in sympatric cattle and badger populations. *PLoS Pathog.* **2012**, *8*.

2. Trewby, H.; Wright, D.; Breadon, E.L.; Lycett, S.J.; Mallon, T.R.; McCormick, C.; Johnson, P.; Orton, R.J.; Allen, A.R.; Galbraith, J.; et al. Use of bacterial whole-genome sequencing to investigate local persistence and spread in bovine tuberculosis. *Epidemics* **2016**, *14*, 26–35.

3. Acosta, F.; Chernyaeva, E.; Mendoza, L.; Sambrano, D.; Correa, R.; Rotkevich, M.; Tarté, M.; Hernández, H.; Velazco, B.; de Escobar, C.; et al. *Mycobacterium bovis* in Panama, 2013. *Emerg. Infect. Dis.* **2015**, *21*, 1059–1061.

4. Glaser, L.; Carstensen, M.; Shaw, S.; Robbe-Austerman, S.; Wunschmann, A.; Grear, D.; Stuber, T.; Thomsen, B. Descriptive epidemiology and whole genome sequencing analysis for an outbreak of bovine tuberculosis in beef cattle and white-tailed deer in northwestern Minnesota. *PLoS One* **2016**, *11*.

5. Broeckl, S.; Krebs, S.; Varadharajan, A.; Straubinger, R.K.; Blum, H.; Buettner, M. Investigation of intra-herd spread of Mycobacterium caprae in cattle by generation and use of a whole-genome sequence. *Vet. Res. Commun.* **2017**, *41*, 113–128.

6. Sandoval-Azuara, S.E.; Muñiz-Salazar, R.; Perea-Jacobo, R.; Robbe-Austerman, S.; Perera-Ortiz, A.; López-Valencia, G.; Bravo, D.M.; Sanchez-Flores, A.; Miranda-Guzmán, D.; Flores-López, C.A.; et al. Whole genome sequencing of *Mycobacterium bovis* to obtain molecular fingerprints in human and cattle isolates from Baja California, Mexico. *Int. J. Infect. Dis.* **2017**, *63*, 48–56.

7. Crispell, J.; Zadoks, R.N.; Harris, S.R.; Paterson, B.; Collins, D.M.; De-Lisle, G.W.; Livingstone, P.; Neill, M.A.; Biek, R.; Lycett, S.J.; et al. Using whole genome sequencing to investigate transmission in a multi-host system: bovine tuberculosis in New Zealand. *BMC Genomics* **2017**, *18*, 180.

8. Patané, J.S.L.; Martins, J.; Castelão, A.B.; Nishibe, C.; Montera, L.; Bigi, F.; Zumárraga, M.J.; Cataldi, A.A.; Junior, A.F.; Roxo, E.; et al. Patterns and processes of *Mycobacterium bovis* evolution revealed by phylogenomic analyses. *Genome Biol. Evol.* **2017**, *9*, 521–535.

9. Dippenaar, A.; Parsons, S.D.C.; Miller, M.A.; Hlokwe, T.; Gey van Pittius, N.C.; Adroub, S.A.; Abdallah, A.M.; Pain, A.; Warren, R.M.; Michel, A.L.; et al. Progenitor strain introduction of *Mycobacterium bovis* at the wildlife-livestock interface can lead to clonal expansion of the disease in a single ecosystem. *Infect. Genet. Evol.* **2017**, *51*, 235–238.

10. Zimpel, C.K.; Brandão, P.E.; Souza Filho, A.F. de; De Souza, R.F.; Ykuta, C.Y.; Soares Ferreira Neto, J.; Soler Camargo, N.C.; Bryan Heinemann, M.; Guimaraes, A.M.S. Complete genome sequencing of *Mycobacterium bovis* SP38 and comparative genomics of *Mycobacterium bovis* and M. tuberculosis strains. *Front. Microbiol.* **2017**, *8*, 2389.

11. Ghebremariam, M.K.; Hlokwe, T.; Rutten, V.P.M.G.; Allepuz, A.; Cadmus, S.; Muwonge, A.; Robbe-Austerman, S.; Michel, A.L. Genetic profiling of *Mycobacterium bovis* strains from slaughtered cattle in Eritrea. *PLoS Negl. Trop. Dis.* **2018**, *12*, e0006406.

12. Kohl, T.A.; Utpatel, C.; Niemann, S.; Moser, I. *Mycobacterium bovis* persistence in two different captive wild animal populations in Germany: A longitudinal molecular epidemiological study revealing pathogen transmission by whole-genome sequencing. *J. Clin. Microbiol.* **2018**, *56*, 1–9.

13. Lasserre, M.; Fresia, P.; Greif, G.; Iraola, G.; Castro-Ramos, M.; Juambeltz, A.; Nuñez, Á.; Naya, H.; Robello, C.; Berná, L. Whole genome sequencing of the monomorphic pathogen *Mycobacterium bovis* reveals local differentiation of cattle clinical isolates. *BMC Genomics* **2018**, *19*, 1–14.

14. Orloski, K.; Robbe-austerman, S.; Stuber, T.; Hench, B.; Schoenbaum, M. Whole genome sequencing of *Mycobacterium bovis* isolated from livestock in the United States, 1989-2018. *Front. Vet. Sci.* **2018**, *5:253*, 1–23.

15. Otchere, I.D.; van Tonder, A.J.; Asante-Poku, A.; Sánchez-Busó, L.; Coscollá, M.; Osei-Wusu, S.; Asare, P.; Aboagye, S.Y.; Ekuban, S.A.; Yahayah, A.I.; et al. Molecular epidemiology and whole genome sequencing analysis of clinical *Mycobacterium bovis* from Ghana. *PLoS One* **2019**, *14*, 1–13.

16. Abdelaal, H.F.M.; Spalink, D.; Amer, A.; Steinberg, H.; Hashish, E.A.; Nasr, E.A.; Talaat, A.M. Genomic Polymorphism Associated with the Emergence of Virulent Isolates of *Mycobacterium bovis* in the Nile Delta. *Sci. Rep.* **2019**, *9*, 1–15.

17. Salvador, L.C.M.; O’Brien, D.J.; Cosgrove, M.K.; Stuber, T.P.; Schooley, A.M.; Crispell, J.; Church, S. V.; Gröhn, Y.T.; Robbe-Austerman, S.; Kao, R.R. Disease management at the wildlife-livestock interface: Using whole-genome sequencing to study the role of elk in *Mycobacterium bovis* transmission in Michigan, USA. *Mol. Ecol.* **2019**, *28*, 2192–2205.

18. Zimpel, C.K.; Patané, J.S.L.; Guedes, A.C.P.; Souza, R.F.; Pereira-Silva, T.T.; Soler Camargo, N.C.; de Souza Filho, A.F.; Ikuta, C.Y.; Soares Ferreira Neto, J.; Setubal, J.C.; et al. Global distribution and evolution of *Mycobacterium bovis*. *bioRxiv* **2019**, 1–25.

19. Michelet, L.; Conde, C.; Branger, M.; Cochard, T.; Biet, F.; Boschiroli, M.L. Transmission Network of Deer-Borne *Mycobacterium bovis* Infection Revealed by a WGS Approach. **2019**, 1–11.

20. Crispell, J.; Benton, C.H.; Balaz, D.; De Maio, N.; Akhmetova, A.; Allen, A.; Biek, R.; Presho, E.L.; Dale, J.; Hewinson, G.; et al. Combining genomics and epidemiology to analyse bi-directional transmission of *Mycobacterium bovis* in a multi-host system. *Elife* **2019**, 1–36.

21. Anzai, E.K. Sequenciamento do genoma completo do *Mycobacterium bovis* como instrumento de sistema de vigilância no Estado de Santa Catarina, University of São Paulo, 2019.

22. Hauer, A.; Michelet, L.; Cochard, T.; Branger, M.; Nunez, J.; Boschiroli, M.-L.; Biet, F. Accurate phylogenetic relationships among *Mycobacterium bovis* strains circulating in France based on whole genome sequencing and single nucleotide polymorphism analysis. *Front. Microbiol.* **2019**, *10*, 955.
