# Supplementary material for: Mycobacterium bovis: From Genotyping to Genome Sequencing
Source: Microorganisms. 2020 May 3;8(5):667. doi: 10.3390/microorganisms8050667 (PMC7285088; doi:10.3390/microorganisms8050667)
Supplement: Supplementary file 1 [file microorganisms-08-00667-s001.zip › Table_S2_correct.docx]

**Table S2.** Characteristics of genotyping techniques and whole-genome sequencing (WGS) used in *Mycobacterium bovis* studies.

| **Methods** | **Principle** | **Minimum DNA input** | **Time** | **Advantages** | **Disadvantages** |
| --- | --- | --- | --- | --- | --- |
| REA | Application of three different restriction enzymes in *M. bovis* total DNA | High (4 -6 μg) | 2 days | - Standardized procedures - Used for strain typing in *M. bovis* | - Too many bands that are hard to resolve - Technically demanding - Requires *M. bovis* isolation |
| PFGE | DNA is digested with rare cutting enzymes and separated by electrical pulses in agarose gel. | High (2 μg) | 7 days | - Can distinguish MTBC species | - Low intra-specific discriminatory power - Labor intensive - Difficult obtaining agarose plugs - Requires intact DNA for restriction enzyme treatment - Requires *M. bovis* isolation - No standardized procedure has been defined - Long turn-over time |
| IS*6110*-RFLP | Digestion of *M. bovis* total DNA with restriction enzyme, followed by electrophoresis in agarose gel and Southern Blot with IS6110 probes | High (2-3 μg) | 3 to 4 days | - Standardized procedures - Indicated for *M. tuberculosis* typing, but not *M. bovis* | - No discriminatory power for *M. bovis* - Labor intensive - Technically demanding - Requires *M. bovis* isolation |
| PGRS-RFLP | Digestion of *M. bovis* total DNA with restriction enzyme, followed by electrophoresis in agarose gel and Southern Blot with PGRS probes | High (2-3 μg) | 3 to 4 days | - Standardized procedures - Used for strain typing in *M. bovis* | - Too many bands that are hard to resolve - Labor intensive - Technically demanding |
| Spoligotyping | PCR-based technique that amplifies the DR locus, followed by hybridization of the biotin-labelled PCR product to a nylon membrane containing oligonucleotides of the DR-related spacers. Spoligotyping targets on single genomic locus. | Extremely low (10 fg) [1] (even from extracted infected tissue) | 2 to 3 days (up to 40 samples) | - Standardized procedures - Can be applied directly to DNA extracted from infected tissue - Automatization - High-throughput - Standardized report - Used for strain typing in *M. bovis* | - Homoplasy - Lower resolution when compared to REA and MIRU-VNTR - Labor intensive |
| MIRU-VNTR PCR | PCR-based amplification of VNTR loci and visualization following gel electrophoresis. MIRU-VNTR PCR targets many genomic loci. | Extremely low (10 fg) [1] (even from extracted infected tissue) | 1 to 2 days for few samples* | - Standardized procedures - Can be applied directly to DNA extracted from infected tissue - Standardized report - Used for strain typing in *M. bovis* | - Variable discriminating power depending on the chosen loci |
| WGS | DNA is digested or sheared, and all fragments are simultaneously sequenced in a next-generation sequencer. The whole genome sequence is available at the end. | Low to high** (1ng to 1ug) | 2 to 4 days*** (~24 to 96 samples) | - Standardized procedures for sequencing - High-throughput - Reproducible - High resolution - Can be used to detect spoligotypes and MIRU-VNTR patterns - Used for strain typing in *M. bovis* - Used for phylogenetic studies of *M. bovis* | - Requires *M. bovis* isolation - Repeats are difficult to resolve using short reads - Technically demanding - Requires more advanced bioinformatic skills - Further standardization of data analysis is needed |

* Time can also vary depending on the number of samples being tested. For instance, one single sample to be run in MIRU-VNTR PCR will be accomplished faster than a spoligotyping. However, spoligotyping can be run with up to 40 samples, and if these same samples are analyzed using MIRU-VNTR PCR, the time spent increases significantly and is dependable on the infrastructure of the laboratory (e.g. availability of large gel electrophoresis systems). ** Total DNA input for WGS (whole-genome sequencing) will depend on the chosen library. There are libraries that can work with low amount of DNA. *** Run time is highly dependable on the chosen protocol. The time of interrupted work for WGS can vary from 19 to 86 hours. Time estimates are approximations given that procedures sometimes need to be performed in different days. The time was also defined without considering bacterial isolation, which can take up to 90 days, depending on the conditions. REA: Restriction endonuclease analysis; PFGE: pulsed-field gel electrophoresis; RFLP: restriction fragment length polymorphism; MIRU-VNTR: mycobacterial interspersed repetitive unit-variable-number tandem repeat typing, polymerase chain reaction.

**References**

1. Kulkarni, S.; Sola, C.; Filliol, I.; Rastogi, N.; Kadival, G. Spoligotyping of *Mycobacterium tuberculosis* isolates from patients with pulmonary tuberculosis in Mumbai, India. *Res. Microbiol.* **2005**, *156*, 588–596.
