# Supplementary material for: Mycobacterium bovis: From Genotyping to Genome Sequencing
Source: Microorganisms. 2020 May 3;8(5):667. doi: 10.3390/microorganisms8050667 (PMC7285088; doi:10.3390/microorganisms8050667)
Supplement: Supplementary file 1 [file microorganisms-08-00667-s001.zip › Figure_S1.pdf]

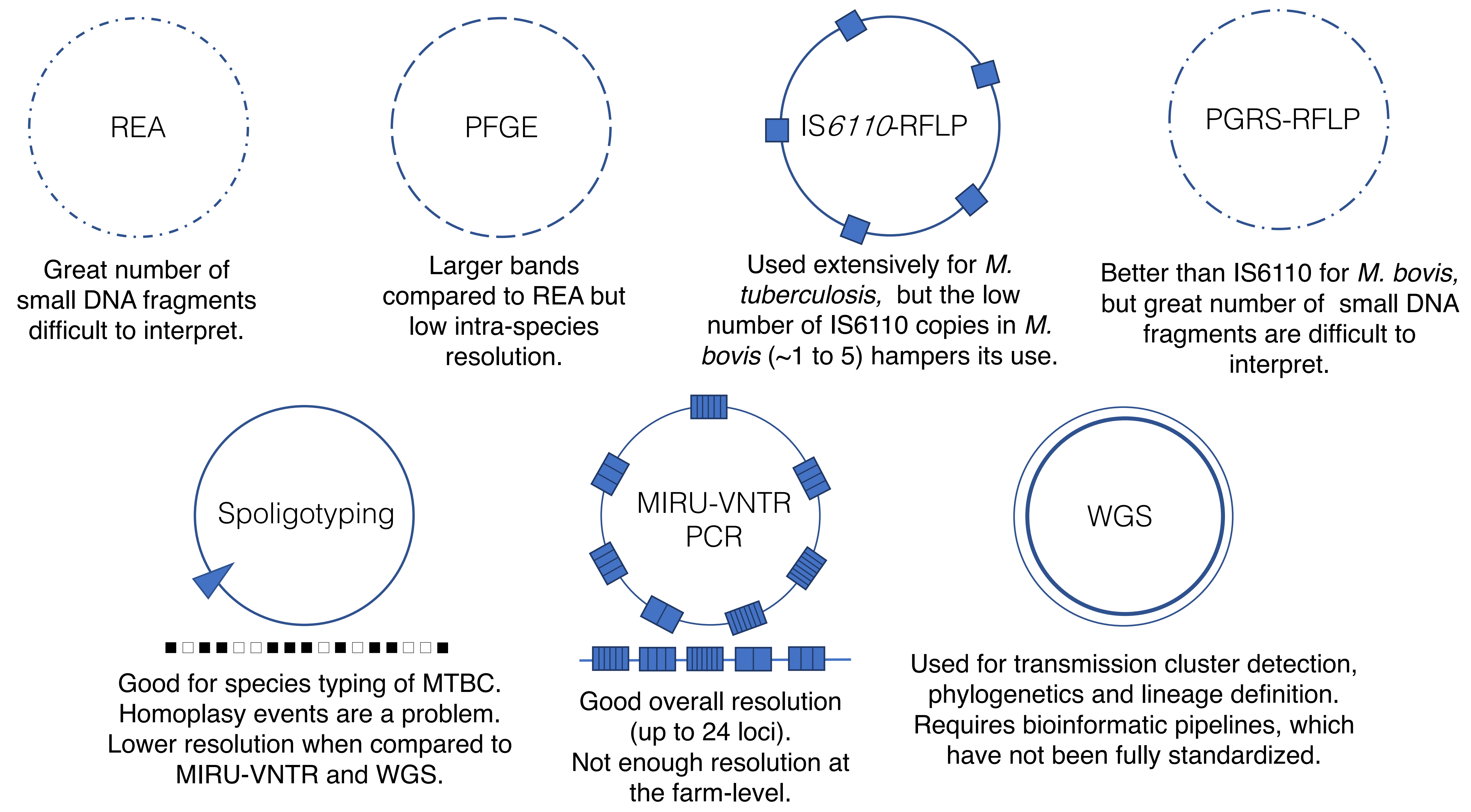

Figure S1. Schematic representation of the genomic regions involved in genotyping techniques used in *Mycobacterium bovis* studies. The main advantage and disadvantage are highlighted in each technique. Many of the disadvantages is what led to the development and application of more sophisticated techniques, until reaching WGS. REA: restriction endonuclease analysis; PFGE: pulsed-field gel electrophoresis; IS6110-RFLP: insertion sequence 6110, restriction fragment length polymorphism; MIRU-VNTR: MIRU-VNTR: mycobacterial interspersed repetitive unit-variable-number tandem repeat typing; PCR: polymerase chain reaction; WGS: whole-genome sequencing.
